# Methods for Specifying Scientific Data Standards and Modeling Relationships with Applications to Neuroscience

**DOI:** 10.3389/fninf.2016.00048

**Published:** 2016-11-04

**Authors:** Oliver Rübel, Max Dougherty, Prabhat, Peter Denes, David Conant, Edward F. Chang, Kristofer Bouchard

**Affiliations:** ^1^Computational Research Division, Lawrence Berkeley National LaboratoryBerkeley, CA, USA; ^2^Biological Systems and Engineering Division, Lawrence Berkeley National LaboratoryBerkeley, CA, USA; ^3^National Energy Research Scientific Computing Center, Lawrence Berkeley National LaboratoryBerkeley, CA, USA; ^4^Physical Sciences Division, Lawrence Berkeley National LaboratoryBerkeley, CA, USA; ^5^Neuroscience, University of California, San Francisco Medical Center, University of California, San FranciscoSan Francisco, CA, USA

**Keywords:** data format specification, relationship modeling, electrophysiology, neuroscience

## Abstract

Neuroscience continues to experience a tremendous growth in data; in terms of the volume and variety of data, the velocity at which data is acquired, and in turn the veracity of data. These challenges are a serious impediment to sharing of data, analyses, and tools within and across labs. Here, we introduce BRAINformat, a novel data standardization framework for the design and management of scientific data formats. The BRAINformat library defines application-independent design concepts and modules that together create a general framework for standardization of scientific data. We describe the formal specification of scientific data standards, which facilitates sharing and verification of data and formats. We introduce the concept of *Managed Objects*, enabling semantic components of data formats to be specified as self-contained units, supporting modular and reusable design of data format components and file storage. We also introduce the novel concept of *Relationship Attributes* for modeling and use of semantic relationships between data objects. Based on these concepts we demonstrate the application of our framework to design and implement a standard format for electrophysiology data and show how data standardization and relationship-modeling facilitate data analysis and sharing. The format uses HDF5, enabling portable, scalable, and self-describing data storage and integration with modern high-performance computing for data-driven discovery. The BRAINformat library is open source, easy-to-use, and provides detailed user and developer documentation and is freely available at: https://bitbucket.org/oruebel/brainformat.

## 1. Introduction

Neuroscience research is facing an increasingly challenging data problem due to the growing complexity of experiments and the volume/variety of data being collected from many acquisition modalities. Neuroscientists are routinely collecting data in a broad range of formats that are often highly domain specific, *ad-hoc* and/or designed for efficiency with respect to very specific tools and data types. Even for single experiments, scientists are interacting with often tens of different formats—one for each recording device and/or analysis—while many formats are not well-described or are only accessible via proprietary software. Navigating this quagmire of formats hinders efficient analysis, data sharing, and collaboration and can lead to errors and misinterpretations. File formats and standards that can represent neuroscience data and make the data easily accessible play a key role in enabling scientific discovery, development of reusable tools for analysis, and progress toward fostering collaboration in the neuroscience community.

The requirements toward a data format standard for neuroscience are highly complex and go far beyond the needs of traditional, modality-specific formats (e.g., image, audio, or video formats). A neuroscience data format needs to support the management and organization of large collections of data from many modalities and sources, e.g., neurological recordings, external stimuli, recordings of external responses and events (e.g., motion-tracking, video, audio, etc.), derived analytic results, and many others. To enable data interpretation and analysis, the format needs to also support storage of complex metadata, such as, descriptions of recording devices, experiments, or subjects among others.

In addition, a usable and sustainable neuroscience data format needs to satisfy many technical requirements. For example, the format should be self-describing, easy-to-use, efficient, portable, scalable, verifiable, extensible, easy-to-share, and support self-contained and modular storage. Meeting all these complex needs is a daunting challenge. Arguably, the focus of a neuroscience data standard should be on addressing the application-centric needs of organizing scientific data and metadata, rather than on reinventing file storage methods. We here focus on the design of a framework for standardization of data formats while utilizing HDF5 as the underlying data model and storage format. Using HDF5 has the advantage that it already satisfies most of the basic, technical format requirements; HDF5 is self-describing, portable, extensible, widely supported by programming languages and analysis tools, and is optimized for storage and I/O of large-scale scientific data.

In this manuscript we introduce BRAINformat, a novel data format standardization framework and API for scientific data, developed at the Lawrence Berkeley National Labs in collaboration with neuroscientists at the University of California, Berkeley and the University of California, San Francisco. BRAINformat supports the formal specification and verification of scientific data formats and supports the organization of data in a modular, extensible, and reusable fashion via the concept of *managed objects* (Section 3.1). We introduce the novel concept or *relationship attributes* for modeling of direct relationships between data objects. Relationship attributes support the specification of structural and semantic links between data, enabling users and developers to formally document and utilize object-to-object relationships in a well-structured and programmatic fashion (Section 3.2). We demonstrate the use of chains of object-to-object relationships to model complex relationships between multi-dimensional arrays based on data registration via the concept of advanced *index map relationships* (Section 3.2.4). We demonstrate the application of our framework to design and implement a standard format for electrophysiology data and show how data standardization and relationship-modeling facilitate multi-modal data analysis and data sharing (Section 4).

## 2. Background and related work

The scientific community utilizes a broad range of data formats. Basic formats explicitly specify how data is laid out and formatted in binary or text data files (e.g., CSV, BOF, etc). While such basic formats are common, they generally suffer from a lack of portability, scalability and a rigorous specification. For text-based files, languages and formats, such as the Extensible Markup Language (XML) (Bray et al., 2008) or the JavaScript Object Notation (JSON) (JSON, 2015), have become popular means to standardize documents for data exchange. XML, JSON and other text-based standards (in combination with character-encoding schema, e.g., ASCII or Unicode) play a critical role in practice in the exchange of usually relatively small, structured documents but are impractical for storage and exchange of large scientific data arrays.

For storage of large scientific data, HDF5 (The HDF Group, 2015) and NetCDF (Rew and Davis, 1990) among others, have gained wide popularity. HDF5 is a data model, library, and file format for storing and managing large and complex data. HDF5 supports groups, datasets, and attributes as core data object primitives, which in combination provide the foundation for data organization and storage. HDF5 is portable, scalable, self-describing, and extensible and is widely supported across programming languages and systems, e.g., R, Matlab, Python, C, Fortran, VisIt, or ParaView. The HDF5 technology suite includes tools for managing, manipulating, viewing, and analyzing HDF5 files. HDF5 has been adopted as a base format across a broad range of application sciences, ranging from physics to bio-sciences and beyond (Habermann et al., 2014). Self-describing formats address the critical need for standardized storage and exchange of complex and large scientific data.

Self-describing formats like HDF5 provide general capabilities for organizing data, but they do not prescribe a data organization. The structure, layout, names, and descriptions of storage objects, hence, often still differ greatly between applications and experiments. This diversity makes the development of common and reusable tools challenging. VizSchema (Shasharina et al., 2009) and XDMF (Clarke and Mark, 2007) among others, propose to bridge this gap between general-purpose, self-describing formats and the need for standardized tools via additional lightweight, low-level schema (often based on XML) to further standardize the description of the low-level data organization to facilitate data exchange and tool development.

Application-oriented formats then generally focus on specifying the organization of data in a semantically meaningful fashion, including but not limited to the specification of storage object names, locations, and descriptions. Many application formats build on existing self-describing formats, e.g., NeXus (Klosowski et al., 1997) (neutron, x-ray, and muon data), OpenMSI (mass spectrometry imaging) (Rübel et al., 2013), CXIDB (Maia, 2012) (coherent x-ray imaging), or NetCDF (Rew and Davis, 1990) in combination with CF and COARDS metadata conventions for climate data, and many others. Application formats are commonly described by documents specifying the location and names of data items and often provide application-programmer interfaces (API) to facilitate reading and writing of format files. Some formats are further governed by formal, computer-readable, and verifiable specifications. For example, NeXus uses the NXDL (NeXus International Advisory Committee, 2016) XML-based format and schema to define the nomenclature and arrangement of information in a NeXus data file. On the level of HDF5 groups, NeXus also uses the notion of *Classes* to define the fields that a group should contain in a reusable and extensible fashion.

The critical need for data standards in neuroscience has been recognized by several efforts over the course of the last several years (e.g., Sommer et al., 2016); however, much work remains. Here, our goal is to contribute to this discussion by providing much-needed methods and tools for the effective design of sustainable neuroscience data standards and demonstration of the methods in practice toward the design and implementation of a usable and extensible format with an initial focus on electrocardiography data. The developers of the *Klustakwik* suite (Kadir et al., 2013, 2015) have proposed an HDF5-based data format for storage of spike sorting data. *Orca* (also called *BORG*) (Keith Godfrey, 2014) is an HDF5-based format developed by the Allen Institute for Brain Science designed to store electrophysiology and optophysiology data. The *NIX* (Stoewer et al., 2014) project has developed a set of standardized methods and models for storing electrophysiology and other neuroscience data together with their metadata in one common file format based on HDF5. Rather than an application-specific format, NIX defines highly generic models for data as well as for metadata that can be linked to terminologies (defined via *od*ML) to provide a domain-specific context for elements. The *open metadata Markup Language od*ML (Grewe et al., 2011) is a metadata markup language based on XML with the goal to define and establish an open and flexible format to transport neuroscience metadata. NeuroML (Gleeson et al., 2010) is also an XML-based format with a particular focus on defining and exchanging descriptions of neuronal cell and network models. The Neurodata Without Borders (NWB) (Teeters et al., 2015) initiative is a recent project with the specific goal “*[…] to produce a unified data format for cellular-based neurophysiology data based on representative use cases initially from four laboratories—the Buzsaki group at NYU, the Svoboda group at Janelia Farm, the Meister group at Caltech, and the Allen Institute for Brain Science in Seattle.”* Members of the NIX, KWIK, Orca, BRAINformat, and other development teams have been invited and contributed to the NWB effort. NWB has adopted concepts and methods from a range of these formats, including from the here-described BRAINformat.

## 3. Standardizing scientific data

### 3.1. Data organization and file format API

BRAINformat adopts HDF5 as its main storage backend. HDF5 provides the following primary storage primitives to organize data within HDF5 files:

- **Group:** A group is used—similar to a folder on a file system—to group zero or more storage objects.
- **Dataset:** A dataset defines a multidimensional array of data elements, together with supporting metadata (e.g., shape and data type of the array).
- **Attribute:** Attributes are small datasets that are attached to primary data objects (i.e., groups or datasets) and are used to store additional metadata to further describe the corresponding data object.
- **Dimension Scale:** This is a derived primitive that uses a combination of datasets and attributes to associate datasets with the dimension of another dataset. Dimension scales are used to further characterize dataset dimensions by describing, for example, the time when samples were measured.

Beyond these basic data primitives, we introduce:

- **Relationship Attributes:** Relationship attributes are a novel, custom attribute-type storage primitive that allows us to describe and model structural and semantic relationships between primary data objects in a human-readable and computer-interpretable fashion (described later in Section 3.2).

Neuroscience research inherently relies on complex data collections from many modalities and sources. Examples include neural recordings, audio and video, eye- and motion-tracking, task contingencies, stimuli, analysis results, and many others. It is therefore critically important to specify formats in a modular and extensible fashion while enabling users to easily reuse format modules and integrate new ones. The concept of managed objects allows us to address this central challenge in an easy-to-use and scalable fashion.

#### 3.1.1. Managed objects

A managed object is a primary storage object—i.e., file, group, or dataset—with: (i) a formal, self-contained format specification that describes the storage object and its contents (see Section 3.1.2), (ii) a specific managed type/class, (iii) a human-readable description, and (iv) an optional unique object identifier, e.g., a DOI. In file, these basic managed object descriptors are stored via standardized attributes. Managed object types may be composed—i.e., a file or group may contain other managed objects—and further specialized through the concept of inheritance, enabling the independent specification and reuse of data format components. The concept of managed objects significantly simplifies the file format specification process by allowing larger formats to be specified in an easy-to-manage iterative manner. By encapsulating semantic sub-components, managed objects provide an ideal foundation for interacting with data in a manner that is semantically meaningful to applications.

The BRAINformat library provides dedicated base classes to assist with the specification and development of interfaces for new managed object types. The *ManagedObject* base API implements common features and interfaces to:

- define the specification of a given managed type,
- recursively construct the complete format specification, while automatically resolving nesting of managed objects,
- verify format compliance of a given HDF5 object,
- access common managed object descriptors, e.g., type, description, specification, and object identifier,
- access contained objects, e.g., datasets, groups, managed objects, etc.,
- retrieve all managed objects of a given managed type,
- automatically create manager class instances for HDF5 objects based on their managed type, and
- for creation of new instances of managed objects via a common *create*(..) method.

To implement a new managed object type, a developer simply defines a new class that inherits from the appropriate base type, i.e., *ManagedFile*, *ManagedGroup*, or *ManagedDataset*. Next, the developer implements the class method *get*_*format*_*specification*(…) to create a formal format specification (see Section 3.1.2) and implements the object method *populate*(…) to define the type-specific population of managed storage objects to ensure format compliance upon creation, i.e., the goal is to avoid that managed objects can be created in an invalid state to ensure format compliance throughout their life cycle.

BRAINformat efficiently supports self-contained, modular, and mixed data storage strategies, by allowing managed groups and datasets to be stored either directly within the same file as the parent group or separately in an external HDF5 file and included in the parent via an external link. To transparently support external storage of managed objects, we provide a generic file storage container for managed objects. This strategy enables users to create and interact with managed objects in the same manner independent of whether they are stored internal or external to the current HDF5 file, effectively hiding the complexity of interacting with possibly large numbers of files. Being able to effectively use self-contained and modular storage strategies is critical for management of neuroscience data due to the diversity and large number of measurements and derived data products that need to be managed and analyzed in conjunction. Self-contained storage eases data sharing, as all data is available in one file. Modular storage then allows us to dynamically link and integrate complex data collections without requiring expensive data copies, eases management of file sizes, and reduces the risk for file corruption by minimizing changes to existing files.

#### 3.1.2. Format specification

To enable the broad application and use of data formats, it is critical that the underlying data standard is easy to interpret by application scientists as well as unambiguously specified for programmatic interpretation and implementation by developers. Therefore, each format component (i.e., managed object type) is described by a formal, self-contained format specification that is computer interpretable while at the same time including human-readable descriptions of all components.

We generally assume that format specifications are minimal, i.e., all file objects that are defined in the specification must adhere to the specification, but a user may add custom objects to a file without violating format compliance. The relaxed assumption of a minimal specification ensures on the one hand that we can share and interact with all format-compliant files and components in a standardized fashion, while at the same time enabling users to easily integrate dynamic and custom data (e.g., instrument-specific metadata). This strategy allows researchers to save all their data using standard format components, even if they only partially cover the specific use-case. This is critical to enable scientists to easily adopt file standards and to allow standards to adapt to the ever-evolving experiments, methods, and use-cases in neuroscience and facilitate new science rather than impeding it.

**Figure 1:**
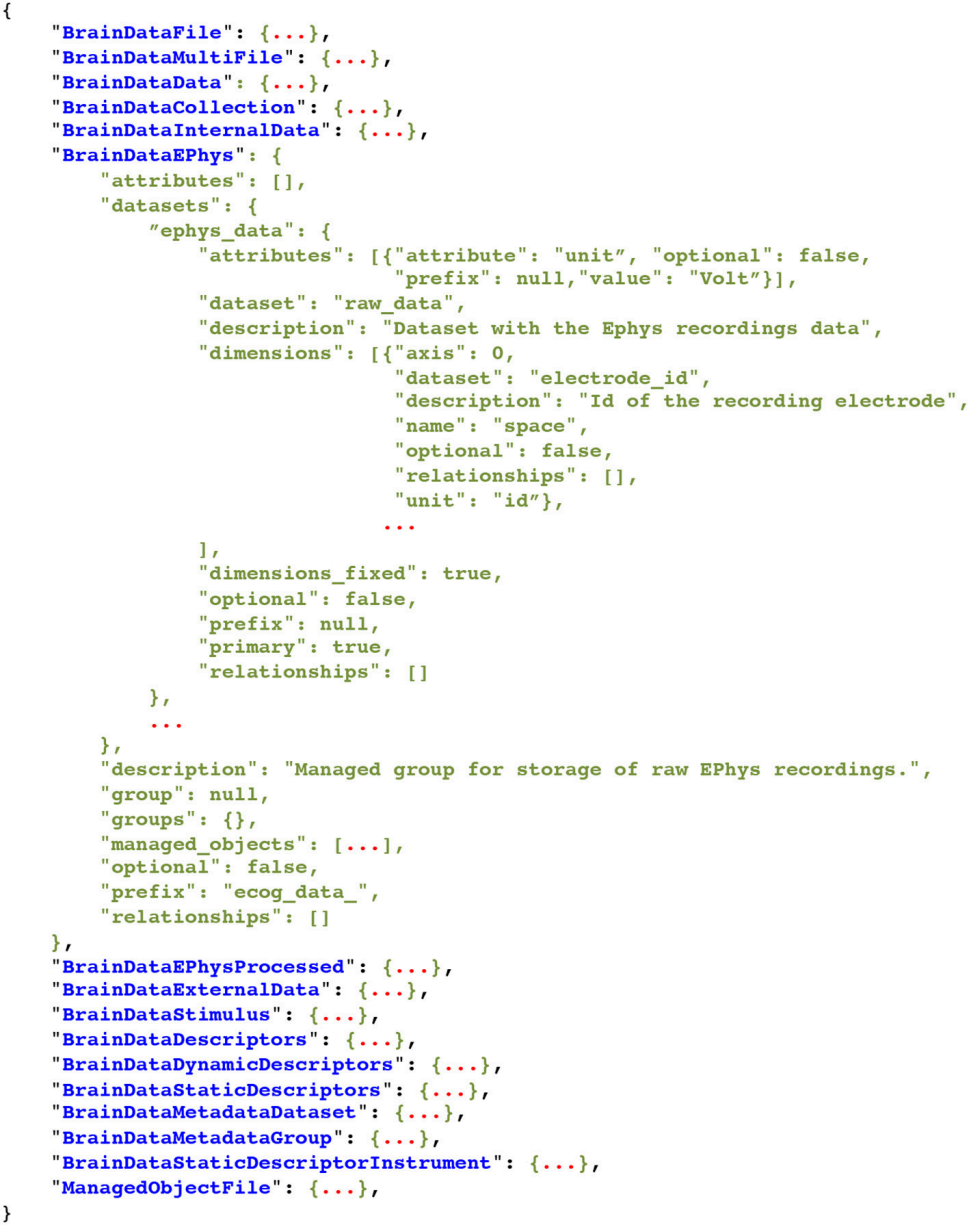
**Abbreviated specification document for our neuroscience data format listing all current managed object types and partial specification for select types, illustrating the general structure of a format specification document**. The full specification document is shown in Supplement 1.3.

The BRAINformat library defines format specification document standards for files, groups, datasets, attributes, dimensions scales, managed objects, and relationship attributes. All specification documents are based on hierarchical Python dictionaries that can be serialized as JSON documents for persistent storage and sharing. For all data objects we specify the name and/or prefix of the object, whether the object is optional or required, and provide a human-readable textual description of the purpose and content of the object. Depending on the object type (e.g., file, group, dataset, or attribute) additional information is specified, e.g., (i) the datasets, groups, and managed objects contained in a group or file, (ii) attributes for datasets, groups and files, (iii) dimension scales of datasets, (iv) whether a dataset is a primary dataset for visualization and analysis or (v) relationships between objects. Figure 1 shows as an example an abbreviated summary of the format specification of our example electrophysiology data standard described in Section 4 and Supplement 1. Relationship attributes and their specification are discussed further in Section 3.2.

The BRAINformat library implements a series of dedicated data structures to facilitate development of format specifications, to ensure their validity, and to simplify the use and interaction with format documents. Using these data structures enables the incremental creation of format specifications, allowing the developer to step-by-step define and compose format specifications. The process of creating new format specifications is in this way much like creating an HDF5 file, making the overall process easily accessible to new users. For example, the following simple code can be used to generate the parts of the *BrainDataEphys* specification shown in Figure 1:


  >>> from brain.dataformat.spec import  *
  >>> **# Define the group**
  >>> brain_data_ephys = **GroupSpec(**group=None, prefix=’ephys_data_’,
                                  description=“Managed group for storage of raw Ephys recordings.”**)**
  >>> **# Define the raw dataset and associated attribute and dimension**
  >>> raw_data_spec = **DatasetSpec(**dataset=’raw_data’, prefix=None, optional=False, primary=’True’,
                                  description=“Dataset with the Ephys recordings data”**)**
  >>> raw_data_spec.add_attribute**(** **AttributeSpec(**attribute=’unit’, prefix=None, value=’Volt’**)**  )
  >>> raw_data_spec.add_dimension**(** **DimensionSpec(**name=’space’, unit=’id’, dataset=’electrode_id’,
                                                axis=0, description=“Id of the recording electrode”**)** )
  >>> **# Add the dataset to the group**
  >>> brain_data_ephys.add_dataset(raw_data_spec, ’ephys_data’)
 


Using our specification infrastructure we can easily compile a complete data format specification document that lists all managed object types and their format. The following simple example code compiles the specification document for our neuroscience data format directly from the file format API (see also Section 4):


  >>> from brain.dataformat.spec import FormatDocument
  >>> import brain.dataformat.brainformat as brainformat
  >>> **json_spec = FormatDocument.from_api(module_object=brainformat).to_json()**


Figure 1 shows an abbreviated summary of the result of the above code. Alternatively, we can also recursively construct the complete specification for a given managed object type, e.g., via:


  >>> from brain.dataformat.brainformat import BrainDataFile
  >>> from brain.dataformat.spec import *
  >>> **format_spec = BrainDataFile.get_format_specification_recursive()**  # Construct the document
  >>> **file_spec = BaseSpec.from_dict(format_spec)**                 # Verification of the document
  >>> **json_spec = file_spec.to_json(pretty=True)**                  # Convert the document to JSON


In this case, all references to other managed objects are automatically resolved and their specification embedded in the resulting specification document. While the basic specification for *BrainDataFile* consists only of ≈11 lines of code (see Supplement 1.1.2), the full, recursive specification contains more than 2170 lines (see Supplement 1.2). Being able to incrementally define format specifications is critical because it allows us to easily extend the format in a modular fashion, define and maintain semantic subcomponents in a self-contained fashion, and avoids hard-to-maintain, monolithic, large documents while still making it easy to create comprehensive specifications documents when necessary.

The ability to compile complete format specification documents directly from data format APIs allows developers to easily integrate new format components (i.e., managed object types) in a self-contained fashion simply by adding a new API class without having to maintain separate format specification documents. Furthermore, this strategy avoids inconsistencies between data format APIs and specification documents since format documents are updated automatically.

The concept of managed objects in combination with the format specification language and API provide an application-independent design concept that allows us to define application-specific formats and modules that are built on best practices.

### 3.2. Modeling data relationships

Neuroscience data analytics often rely on complex structural and semantic relationships between datasets. For example a scientist may use audio recordings to identify particular speech events during the course of an experiment and in turn needs to locate the corresponding data in an electrocorticography recording dataset to study the neural response to the events. In addition, we often encounter structural relationships in data, for example, when using index arrays or when datasets have been acquired simultaneously and/or using the same recording device and many others. To enable efficient analysis, reuse, and sharing of neuroscience data it is critical that we can model the diverse relationships between data objects in a structured fashion to enable human and computer discovery, use, and interpretation of relationships.

Modeling data relationships is not well-supported by traditional data formats, but is typically closer to the domain of scientific databases. In HDF5, we can compose data via HDF5 links (soft and hard) and associate datasets with the dimensions of another dataset via the concept of dimension scales. However, these concepts are limited to very specific types of data links that do not describe the semantics of the relationship. A new general approach is needed to describe more complex structural and semantic links between data objects in HDF5.

#### 3.2.1. Specifying and storing relationships

Here we introduce the novel concept of *relationship attributes* to describe complex semantic relationships between a source object and a target data object in a general and extensible fashion. Relationship attributes are associated with the source object and describe how the source is related to the target data object. The source and target of a relationship may be either a HDF5 group or dataset.

**Figure 2:**
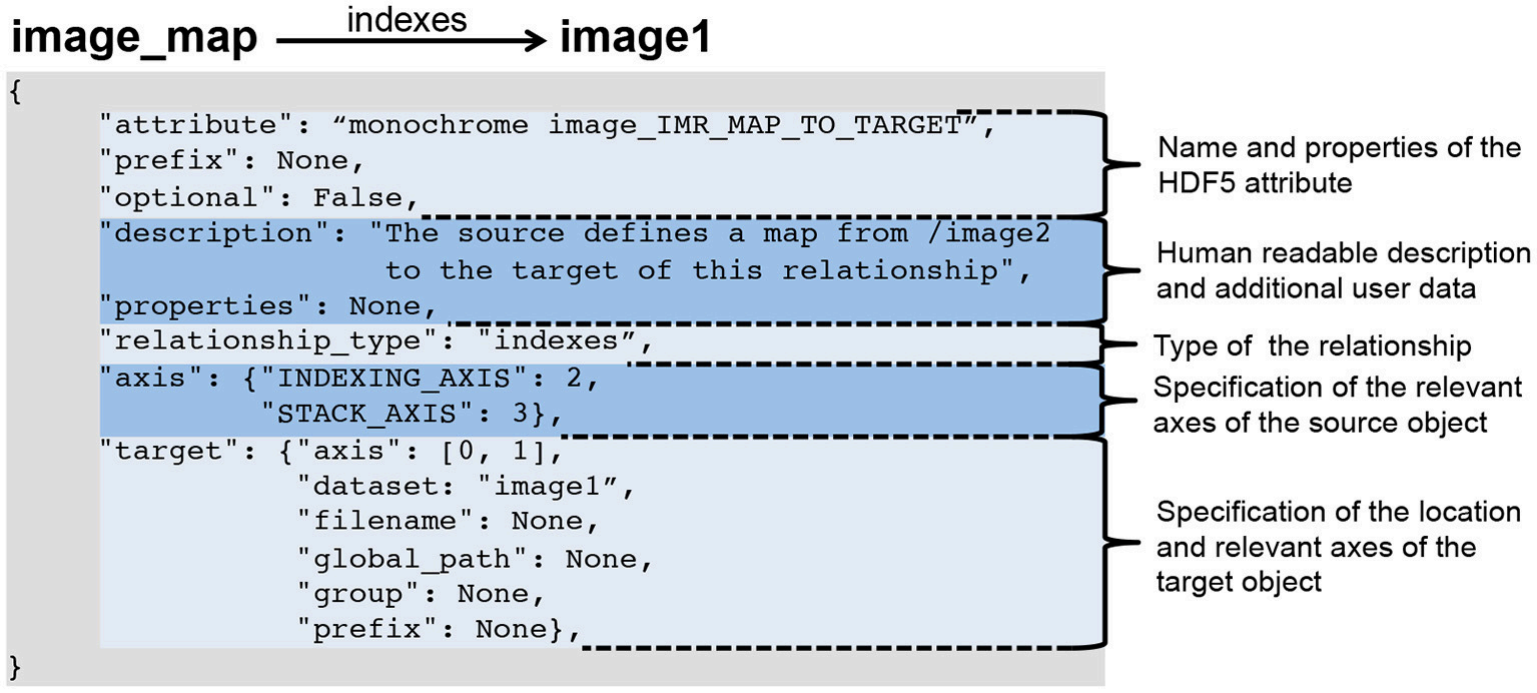
**Example specification of a relationship attribute**.

Relationship attributes are like other file components specified via a JSON dictionary and are part of the specification of datasets and groups. Like any other data object, relationships may also be created dynamically to describe relationships that are unknown *a priori*. Specific instances of relationships are stored as attributes on the source HDF5 object, where the value of the attribute is the JSON document describing the relationship. As illustrated in Figure 2, the JSON specification of a relationship consists of:

- The specification of the name of the attribute and whether the attribute is optional. When stored in HDF5 we prepend the prefix *RELATIONSHIP*_*ATTR*_ to the user-defined name of the attribute to describe the attribute's class and ease identification of relationship attributes.
- A human-readable description of the relationship and an optional JSON dictionary with additional user-defined metadata.
- The specification of the type of the relationship (described next in Section 3.2.2).
- The specification of the axes of the source object to which the relationship applies. This may be: (i) a single index, (ii) a list of axes, (iii) a dictionary of axis indices if the axes have a specific user meaning, or iv) None if the relationship applies to the source object as a whole. Note, we do not need to specify the location of the source object, as the specification of the relationship is always associated with either the source object in HDF5 itself or in the format specification.
- The specification of the target object describing the location of the object and the axes relevant to the relationship (using the same relative ordering or names of axes as for the source object).

#### 3.2.2. Relationship types

The relationship type describes the semantic nature of the relationship. The BRAINformat library currently supports the following relationship types, while additional types can be added in the future:

- **order:** This type indicates that elements along the specified axes of the relationship are ordered in the target in the same way as in the source. This type of relationship is very common in practice. For example, in the case of dimension scales an implicit assumption is that the ordering of elements along the first axis of the scale-dataset matches the ordering of the elements of the dimension it describes. This assumption, however, is only implicit and is by no means always true (nor does HDF5 require this relationship to be true). Using an *order* relationship we can make this relationship explicit. Other common uses of *order* relationships include describing the matched ordering of electrodes in datasets that have been recorded using the same device or matched ordering of records in datasets that have been acquired synchronously.
- **equivalent:** This type expresses that the source and target object encode the same data (even if they might store different values). This relationship type also implies that the source and target contain the same number of values ordered in the same fashion. This relationship occurs in practice any time the same data is stored multiple times with different encodings. For example, to facilitate data processing a user may store a dataset of strings with the names of tokens and store another dataset with the equivalent integer ID of the tokens (e.g., |*baa*| = 1, |*gaa*| = 2, etc.).
- **indexes:** This type describes that the source dataset contains indices into the target dataset or group. In practice this relationship type is used to describe basic data structure where we store, for example, a list of unique values (tokens) along with other arrays that reference that list.
- **shared encoding:** This type indicates that the source and target data object contain values with the same encoding so that the values can be directly compared via equals “==”. This relationship is useful in practice any time two objects (datasets or groups) contain data with the same encoding (e.g., two datasets describing external stimuli using the same ontology).
- **shared ascending encoding:** This type implies that the source and target object share the same encoding and that the values are sorted in ascending order in both. The additional constraint on the ordering enables i) comparison of values via greater than “>” and less than “<” in addition to equals == and ii) more efficient processing and comparison of data ranges. For example, in the case of two datasets that encode *time*, we often find that individual time points do not match exactly between the source and target (e.g., due to different sampling rates). However, due to the ascending ordering of values, a user is still able to compare ranges in *time* in a meaningful way.
- **indexes values:** This type is typically used to describe value-based referencing of data and indicates that the source object selects certain parts of the target based on data values (or keys in the case of groups). This relationship is a special type of *shared encoding* relationship.
- **user:** The *user* relationship is a general container to allow users to specify custom semantic relationships that do not match any of the existing relationship patterns. Additional metadata about the relationship may be stored as part of the user-defined *properties* dictionary of the relationship attribute.

#### 3.2.3. Using relationship attributes

Relationship attributes are a direct extension to the previously described format specification infrastructure. Similar to other main data objects, BRAINformat provides dict-like data structures to help with the formal specification of relationship attributes. In addition, the BRAINformat library also provides a dedicated *RelationshipAttribute* API, which supports creation and retrieval of relationship attributes (as well as index map relationship, described in Section 3.2.4) and provides easy access to the source and target HDF5 object and corresponding specifications of relationship attributes.

**Figure 3:**
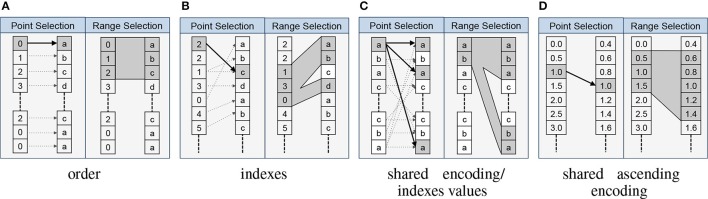
**Overview of the main relationship types and the implied mapping of point- and range-based selections from the source to the target object**. In each cell we show the source object on the left and the target object of the relationship on the right. **(A)** For *order* relationships we can directly map array indices between the data objects. In the case of *order* relationships involving HDF5 Groups we assume alphabetic ordering of elements. **(B)** In the case of *indexes* relationships we map selections by retrieving the relevant indices from the source array. **(C)** For *shared encoding* and *indexes values* relationships we support data selection via value-based data mapping, i.e., we map selections by locating all data values in the target object that match at least one of the values we selected in the source object. **(D)** *Shared ascending encoding* relationships behave in general similar to *shared encoding* relationships, however, the additional constraint that values are sorted in ascending order enables us to map range selections directly based on the minimum and maximum value selected in the source (in contrast to the strict equal value matching of *shared encoding*). **(E)** *User* relationships define custom user semantics and do not imply a specific mapping between data elements (not shown).

One central advantage of explicitly modeling relationships is that it allows us to formalize the interactions and collaborative usage of related data objects. In particular, the relationship types imply formal rules for how to map data selections from the source to the target of a relationship. The *RelationshipAttribute* API implements these rules and supports standard data selection operations, which allows us to easily map selections from the source to the target data object using a familiar array syntax. For example, assume we have two datasets *A* and *B* that are related via an *indexes* relationship *R*_*A*→*B*_. A user now selects the values *A*[1:10] and wants to locate the corresponding data values in *B*. Using the BRAINformat API we can now simply write *R*_*A*→*B*_[1:10] to map t he selection [1:10] from the source *A* to the target *B*, and if desired retrieve the corresponding data values in *B* via *B*[*R*_*A*→*B*_[1:10]]. Figure 3 provides an overview of the rules for mapping data selections based on the relationship type.

Relationship attributes standardize the specification, storage, and programmatic interface for creating, discovering, and using relationships and related data objects. Describing relationships between data explicitly greatly simplifies the process of interacting with multiple datasets and facilitates the collaborative use of data by enabling utilization of multiple datasets in conjunction without having to *a priori* know the relationships and datasets involved. In this way, relationship attributes also open the route for the standardized development of novel data-driven analytics and workflows based on the programmatic discovery and use of related data objects.

#### 3.2.4. Index map relationships

Beyond the description of direct object-to-object relationships, relationship attributes also form the building blocks that allow us to specify higher-order relationships. Using relationship attributes we can define chains of object-to-object relationships that, when interpreted in conjunction, express highly complex structural and semantic relationships. Imagine the following situation: scientists have acquired an optical microscopy image *A* and a mass spectrometry image (MSI) *B* of the same brain. Using the optical image a scientist identifies a particular brain region of interest and now wants to study the chemical makeup of the same region further using the MSI image. This seemingly simple task of accessing corresponding data values in two related datasets is in practice, however, often highly complex. Even if the data registration problem between the datasets is solved, a user still has to know exactly: (i) the location of both datasets *A* and *B*, (ii) how the two datasets are related, (iii) what the transformations generated by the data registration process are, (iv) how to utilize that information to map between *A* and *B*, and (v) write complex, custom code to access the data.

*Index map relationships* allow us to explicitly describe this complex relationship between *A* and *B* via a simple chain of object-to-object relationship attributes and to greatly simplify the cooperative interaction with the data. Rather than describing the relationship between *A* and *B* directly, users can create an intermediate index map *M*_*A*→*B*_ that stores for each pixel in *A* the index of the corresponding pixel(s) in *B*. *M*_*A*→*B*_ explicitly and unambiguously describes the mapping from *A* to *B* so that we can directly utilize the mapping without having to perform complex and error-prone index transformations (which would be needed if we described the mapping implicitly, e.g., via scaling, rotation, morphing and other data transformations). As Table 1 shows, we can unambiguously describe the complex relationship between *A* and *B* via *M*_*A*→*B*_ through a series of simple relationship attributes.

**Table 1:** **Overview of the relationships used to define an advanced *index map relationship***.

	**Source**	**Relationship**	**Target**	**Description**
1.	*A*	$\overset{order}{\to }$	*M*_*A*→*B*_	This relationship describes that elements in *A* are ordered in the same way as the elements in the index map *M*_*A*→*B*_. In addition we may further specify the axes in the source *A* and target *M*_*A*→*B*_ along which the relationship applies.
2.	*A*	$\overset{order}{\leftarrow }$	*M*_*A*→*B*_	Inverse of (1), describing the object ordering relationship between *M*_*A*→*B*_ and *A*.
3.	*M*_*A*→*B*_	$\overset{indexes}{\to }$	*B*	This relationship indicates that *M*_*A*→*B*_ stores indices into *B* and describes how our map can be used to access *B*. An example specification of this relationship is shown in Figure 2.
4.	*A*	$\overset{user}{\to }$	*B*	An optional user-type relationship may be used to further characterize the semantic relationship between *A* and *B*.

**Figure 4:**
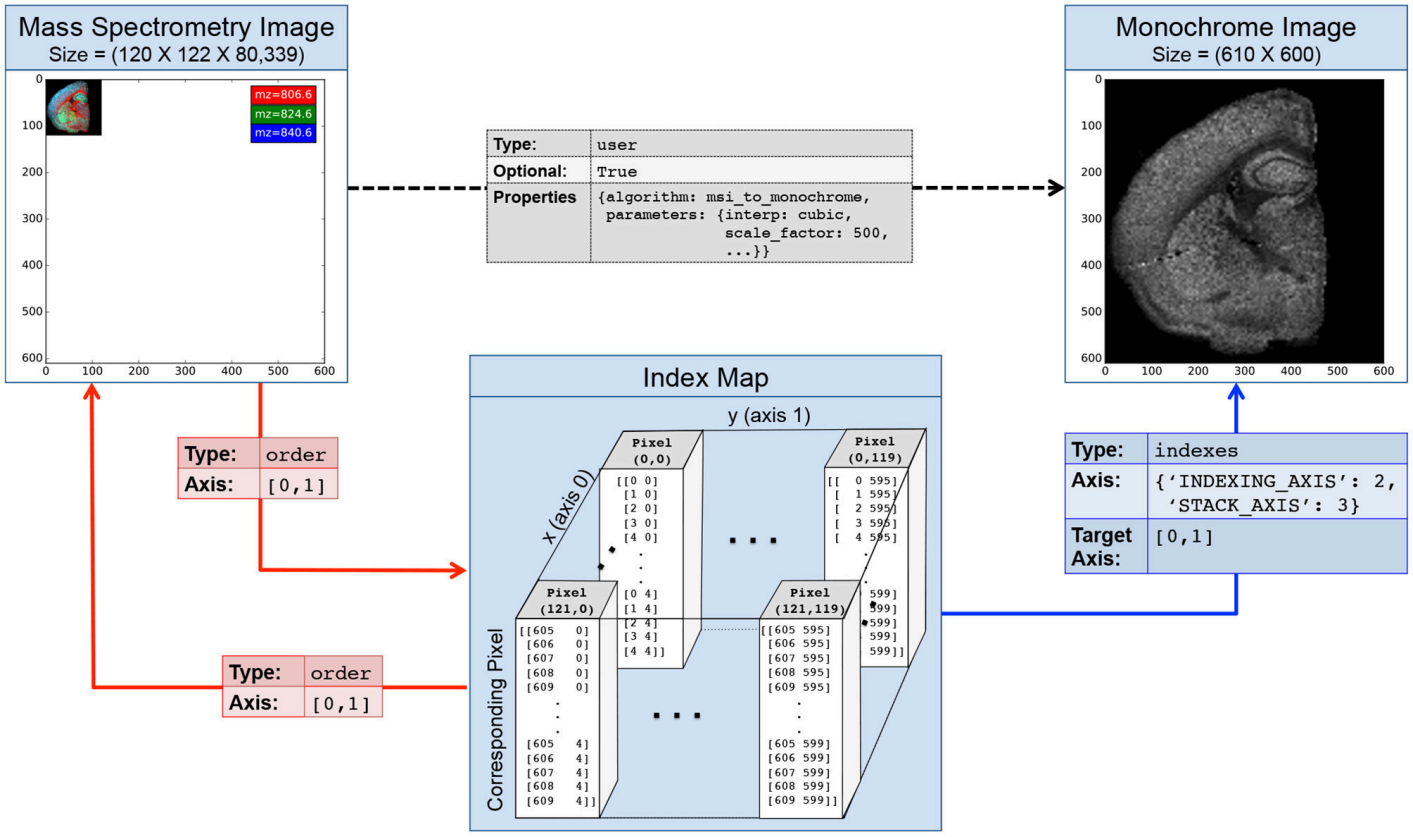
**Illustration of an index map relationship describing the interaction between a mass spectrometry image (MSI) and a derived monochrome image**. The MSI image is in this case 5 × smaller than the monochrome image. The intermediary index map describes for each pixel in the MSI image which pixels it corresponds to in the the monochrome image. Two *order* relationships (red arrows) describe the interactions between the MSI image and the map and *vice versa*. A third *indexes* relationship links our index map to the monochrome image and describes how the map can be used to access the image. Optionally, we may create a fourth *user* relationship (black arrow) to further characterize the semantic relationship between the derived and original image (e.g., to store a description of the algorithm and parameters used to generate the image). Naturally, we can also describe the inverse mapping between the original and processed image via a second index map relationship.

We present a specific example later in Figure 4.

Given only our source dataset *A* (or index map *M*_*A*→*B*_) we can now easily discover all relevant data objects (*A*, *B*, and *M*_*A*→*B*_) and relationships (Table 1) without having to *a priori* know the mapping or the location of the datasets. Via the index map relationship we can now directly map selections: (i) from *A* to *M*_*A*→*B*_ and *vice versa* (ii) from *M*_*A*→*B*_ to *B*, and most importantly (iii) from *A* to *B* simply by selecting data via our *indexes* relationship (Table 1, row 3) to retrieve the corresponding indices from our index map *M*_*A*→*B*_. As data mappings are described explicitly, index map relationships enable registration and mapping under arbitrary transformations. Also, mappings are not required to be unique—i.e., arbitrary N-to-M mappings between elements are permitted—and the source and target of relationships may not just be datasets but also groups, i.e., index map relationship can be used to define mappings between contents of groups or even groups and datasets in HDF5.

BRAINformat implements the concept of index map relationships—similar to dimension scales and relationship attributes—via a set of simple naming conventions for the attribute names. In addition to the *RELATIONSHIP_ATTR* prefix, we use a set of reserved post-fix values for the attribute name to identify the different components of the index map relationship, specifically, *_IMR_SOURCE_TO_MAP, _IMR_MAP_TO_SOURCE, _IMR_MAP_TO_TARGET, _IMR_SOURCE_TO_TARGET*. The BRAINformat API directly supports index map relationships so that we can, for example, directly create and locate all relationships that define an index map relationship via a single function call and programmatically interact with the relationships. The Jupyter notebook available at http://tinyurl.com/jsuzvar provides a tutorial of the Python API for creating and using relationship attributes and index map relationships.

Index map relationships have broad practical applications, including data registration, multi-modal data analysis, sub-component analysis, correlation and alignment of data dimensions, or multi-resolution data storage. Index map relationships are directly applicable to specify the mapping between images in a time series or a stack of physical slices as well as to define correspondences between images from different modalities. We may also define mappings between select dimensions of a dataset to correlate data from different recordings in time or space. Furthermore, analytics are often based on characteristic sub-components of a dataset. As such, a user may extract and separately process sub-components of datasets (e.g., a sub-image of a single cell) and use index map relationships to map the extracted or derived analysis data back to the original data. To optimize data classification and other compute-intensive analyses, a user may perform initial calculations on lower-resolution datasets and use index map relationships to access corresponding data values in high-resolution variants of the data for further processing.

**Figure 5:**
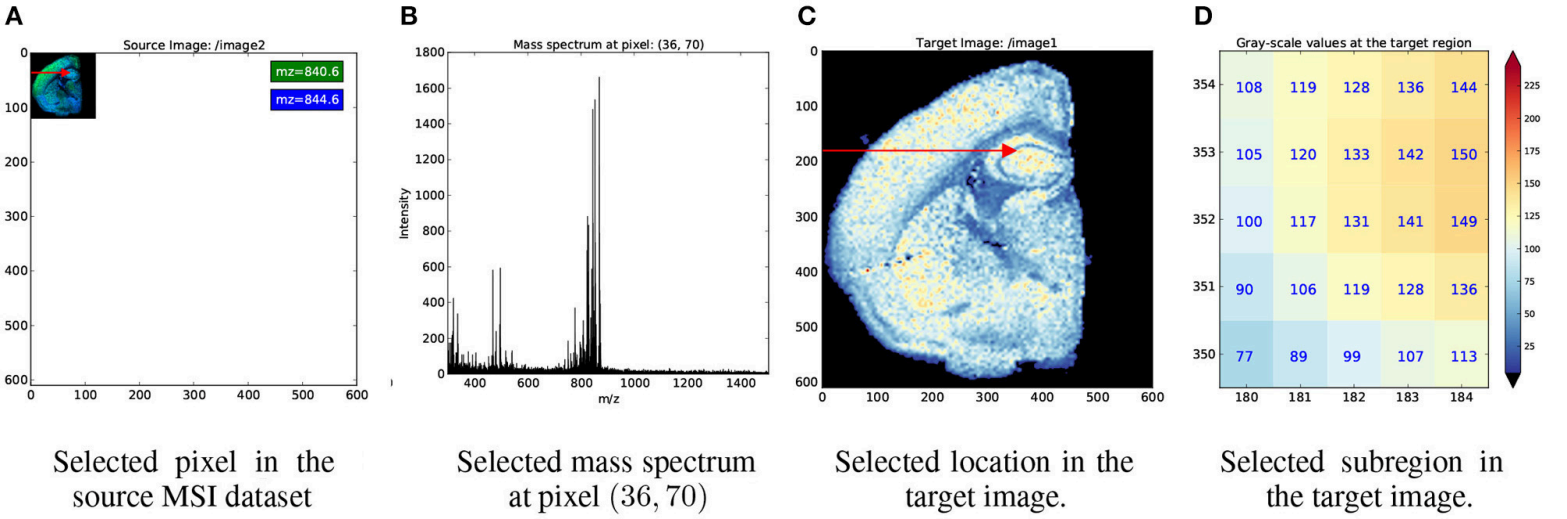
**Example showing the application of index map relationships for data selection. (A)** First we apply the selection [36, 70] (red arrow) to our source, MSI dataset. **(B)** As expected, this results in the selection of a single mass spectrum. **(C)** We next map the same selection to our target dataset. From the red arrow we can see that the selection was mapped correctly to same relative location as in our source, image. **(D)** The pixel plot illustrates that the mapping resulted, as expected, in the selection of a 5 × 5 sub-image from our target image. In **(C,D)** we use the color map shown on the right to map gray-scale values in the monochrome image to color.

Figure 4 illustrates an example index map relationship for mapping between a mass spectrometry imaging (MSI) dataset of the left coronal hemisphere of a mouse brain and a derived, monochrome image. For the example we use an MSI dataset made available by Louie et al. (Lee et al., 2012) via OpenMSI (Rübel et al., 2013). The MSI dataset has a size of (120 × 122 × 80, 339) and has been processed via peak detection and integration, principle component analysis, and interpolation to generate a (610 × 600) monochrome summary image for exploration. Each pixel in the MSI image maps to a 5 × 5 sub-region in the monochrome image. We, hence, create a 4-dimensional index map dataset where: (i,ii) the first two dimensions correspond to the spatial dimensions *x* and *y* of the images, (iii) the third dimension is our index axis of length 2 since each pixel is described by two integer indices, and (iv) the fourth dimension is our stacking axis with the list of all corresponding pixel. Using the BRAINformat API, we can now create the index map relationship (arrows in Figure 4) via a single function call. As illustrated in Figure 5, we can now easily map a selection (here [36, 70]) from our MSI image (source) to the monochrome image (target) simply by applying the selection to our index map relationship (*imr*) via *imr*[′*MAP*_*TO*_*TARGET*′][36, 70] and retrieve the data of the corresponding subimage from our monochrome image. The source code for this example is available at http://tinyurl.com/hvjckhf. In practice the type of interactions illustrated in this example are critical for the analysis of multi-modal imaging experiments, e.g., using high-resolution histology imaging in combination with MS-based chemical imaging.

As this example illustrates, index map relationships allow us to explicitly and unambiguously describe complex data relationships. This is critical to enable programmatic discovery and use of relationships and to perform complex multi-data analytics while reducing risk for errors due to implicit assumptions about relationships. Here we focus on index map relationships, but the same basic concept of chaining relationships could be applied to construct other types of complex object inter-relationships as well.

## 4. Applications to neuroscience data

In the following we describe the application of our framework to develop a format for neuroscience data by focusing on electrophysiology experiments recording from the primary auditory cortex (A1) of anesthetized rats. These data share many requirements with standard physiology data collected by the broader neuroscience community: storage of signal recordings over time across multiple spatially distributed sensors with potentially heterogenous geometries, complex and multi-tiered task descriptions (e.g., complex auditory stimuli), *post-hoc* processing of raw data to extract the signal of interest, the association of physiology data with multi-modal data streams collected simultaneously by other devices (e.g., audio or video recordings of stimuli and subject actions), the linkage of data associated with the same “task” across multiple sessions, and the necessity to store rich metadata to make sense of it all.

### 4.1. High-level data organization

**Figure 6:**
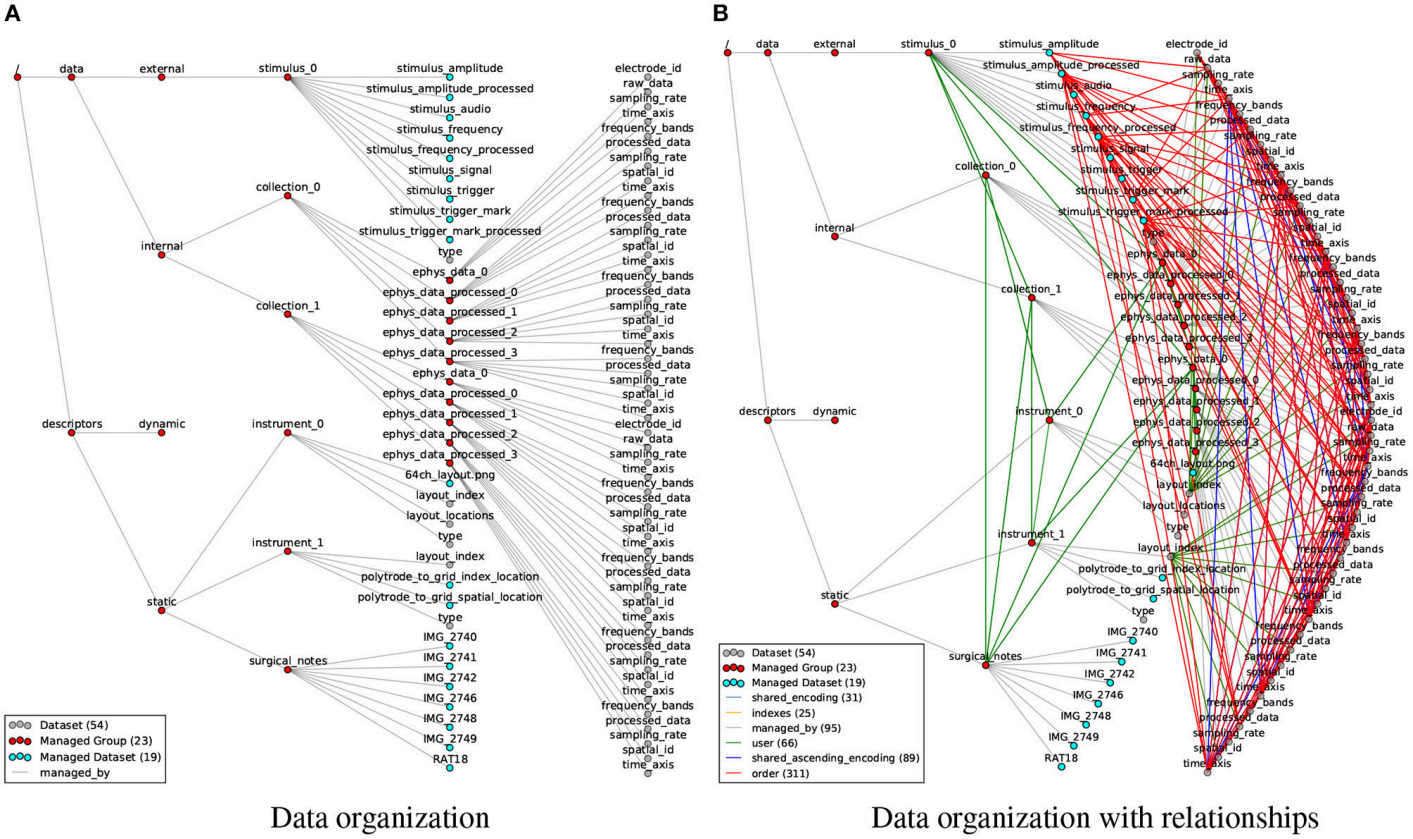
**(A)** Visualization of the organization of datasets and groups in the rat electrophysiology recordings using our data format, with gray edges (*managed by*) indicating the hierarchy of objects in the HDF5 file. **(B)** Same as **(A)** but additionally showing all 522 relationship attributes (colored edges) describing structural and semantic relationships between the data objects. The number of nodes and edges in the graph are indicated in the legend. In addition, the file also contains 372 regular attributes (not shown).

Figure 6A shows the organization of an example electrophysiology dataset from a rat experiment stored using our file format. Scientists in the Bouchard lab recorded neural responses to audio stimuli simultaneously using a micro electrocorticography (μECoG) grid and laminar polytrode. Recordings were subsequently processed for a time-frequency analyses (e.g., wavelet decomposition) and event detection (multi-unit activity). In the file, the data is organized in a series of managed groups with corresponding format specification and API classes. A complete list of all managed object types is shown in Figure 1 and the JSON documents with the complete file and format specification documents are shown in Supplement 1.3 and 1.4.

In the HDF5 file the data is organized in a basic semantic hierarchy. On the highest level we distinguish between data and descriptors, i.e., raw and processed data generated through experimentation and analysis vs. globally accessible metadata. We then further distinguish between static metadata (i.e., descriptions of the basic data acquisition and experimental parameters) and dynamic metadata (e.g., descriptions of post-processing parameters) and categorize data into internal data (i.e., data collected inside the brain, e.g., electrophysiology recordings) and external data (i.e., data collected external to the animal, e.g., sensory stimuli, audio recordings, position of body parts, etc.). Raw and processed data may then either be directly stored as part of the internal and external data groups or further grouped into collections, e.g., here to group data related to the μECoG grid and polytrode, respectively. These divisions impose some minimal structure on the format easing interpretability and traversal by users.

In practice, scientists regularly acquire data in series of distinct experimental sessions often distributed over long periods of time. To facilitate management and sharing of data, it is useful to store the data generated from such distinct recordings in separate data files, yet for analysis purposes the data often needs to be analyzed in context. To allow the organization of related files, we support the grouping of files in managed container files (BrainDataMultiFile) in which each primary file is represented by an HDF5 group /entry_# that defines an external link to the root group of the corresponding file. This simple concept enables users to organize large collections of related files (e.g., data from multiple experimental sessions) and interact with the data as if it were located in a single file. In addition, as described earlier in Section 3.1.1, the BRAINformat library supports modular storage of individual managed objects in separate HDF5 files (which are in turn included in the parent via external links). This allows users to flexibly store and share data from multiple modalities and analytics as independent files while at the same time making the results easily accessible from the main data file and limiting the need for large-scale updates to the main file, significantly reducing risk of data corruption.

### 4.2. Storing electrophysiology data

A common application in neurophysiology experiments is the acquisition and storage of voltage recordings over time across multiple spatially distributed sensors, e.g., via electrocorticography (ECoG), multi-channel electrophysiology from silicon arrays or Utah arrays. The BrainDataEphys module defines a managed group in HDF5 that serves as a container to collect all data pertaining to the voltage recordings by a single device. The primary dataset raw_data defines a two-dimensional, *space* × *time* array storing electrical recordings in units of *Volts*. Auxiliary information about the data, e.g., the sampling_rate in *Hz* among others, are stored as additional datasets and attributes.

The voltage recordings are also further characterized via a series of dimension scales describing: (i) the identifier of electrodes (e.g., linear channel index from DAQ), (ii) the sample time in milliseconds, and optionally (iii) the anatomical name and integer id of the spatial region where each electrode is located. In addition, the BrainDataEphys API allows users to easily add custom dimension scales to the data. Dimension scales are described by: (i) a data array with the scale's data, (ii) the units of the values, (iii) a human-readable description of the contents of the scale, (iv) the name of the scale, and (v) the the associated axis. The ability to easily generate custom dimension scales enables users to conveniently associate additional descriptions with the data, e.g., scales describing the classification of electrodes or time values into unique groups/clusters or to encode the occurrence of different events in time. Dedicated functions for look-up and retrieval of all or select dimensions scales—including all auxiliary data, e.g., the units or description of the scale(s)—ease the integration and use of dimensions scales for analytics.

As described earlier, the creation of managed objects is standardized. All required data structures are initialized during the creation process, ensuring that the file is always valid. Other, optional structures (e.g., the anatomy) may be saved directly during the creation or added later. To ease the use of the format during acquisition, BrainDataEphys supports *auto-expand-data*, a mode in which the raw data and associated dimension scale arrays are initially created as empty datasets that are automatically expanded as new recordings are acquired over time (see also Supplement 2).

A central advantage of the modular managed object design of our format is its reusability and extensibility. For example, in practice, electrophysiology data and other temporal recordings across multiple sensors, are often further processed to extract specific frequency bands or fixed-length events/features (e.g., phonemes or task trials). To support this, the BrainDataEphysProcessed managed type expands BrainDataEphys to support three-dimensional voltage arrays of *space*×*time*×*band* and associated additional metadata and dimension scales. The modular managed object design also greatly simplifies the format specification process by allowing us to define the format incrementally and reduces the size of specification documents. For example, while the full format specification of *BrainDataFile* consists of 2173 lines, the specification of the individual modules is ≈66% shorter (see Supplement 1.2 and 1.3).

Users can interact with the voltage recordings directly via standard array-based data selection (similar to NumPy arrays) while auxiliary data, e.g., the sampling rate and dimensions scales, can be easily retrieved via corresponding access functions or key-based data selection (similar to Python dictionaries). In addition to the rat data described here, we also successfully stored electrocorticography (ECoG) recordings collected from a neurosurgical patient during speech production in the file format (see Supplement 3).

### 4.3. Modeling data relationships

In practice, neuroscience data exhibits complex structural and semantic relationships, e.g., derived datasets and data recorded synchronously or using the same instrument are often related in space and/or time. Unfortunately, data formats typically do not support modeling of data relationships so that users have to infer relationships from the file hierarchy (Figure 6A) and unstructured metadata. In practice, the lack of standardized information about relationships may lead to errors in data analysis and interpretation and hinders efficient data reuse and sharing.

Using the concept of relationship attributes allows us to formalize this process and model complex relationships between datasets in a standardized, human- and computer-readable fashion. Common relationships, e.g., order relationships between dimension scales and actual data and other scales are created automatically by the format library. Custom relationships can then be easily defined via single function calls using the RelationshipAttribute.create(…) API.

Even though the example file in Figure 6A describes just a single recording session with a few additional processed results, the number of relationships quickly exceeds the number of data objects. Modeling only the most common relationships, we established 522 relationship attributes in the rat data example (Figure 6B), describing a highly complex network of links between data objects. It is easy to see why users become quickly overwhelmed when having to remember and tediously reconstruct these data relationships, leading to critical errors and high cost for analysis and hindering the design of reusable, multi-modal analytics.

### 4.4. Multi-modal data analysis

**Figure 7:**
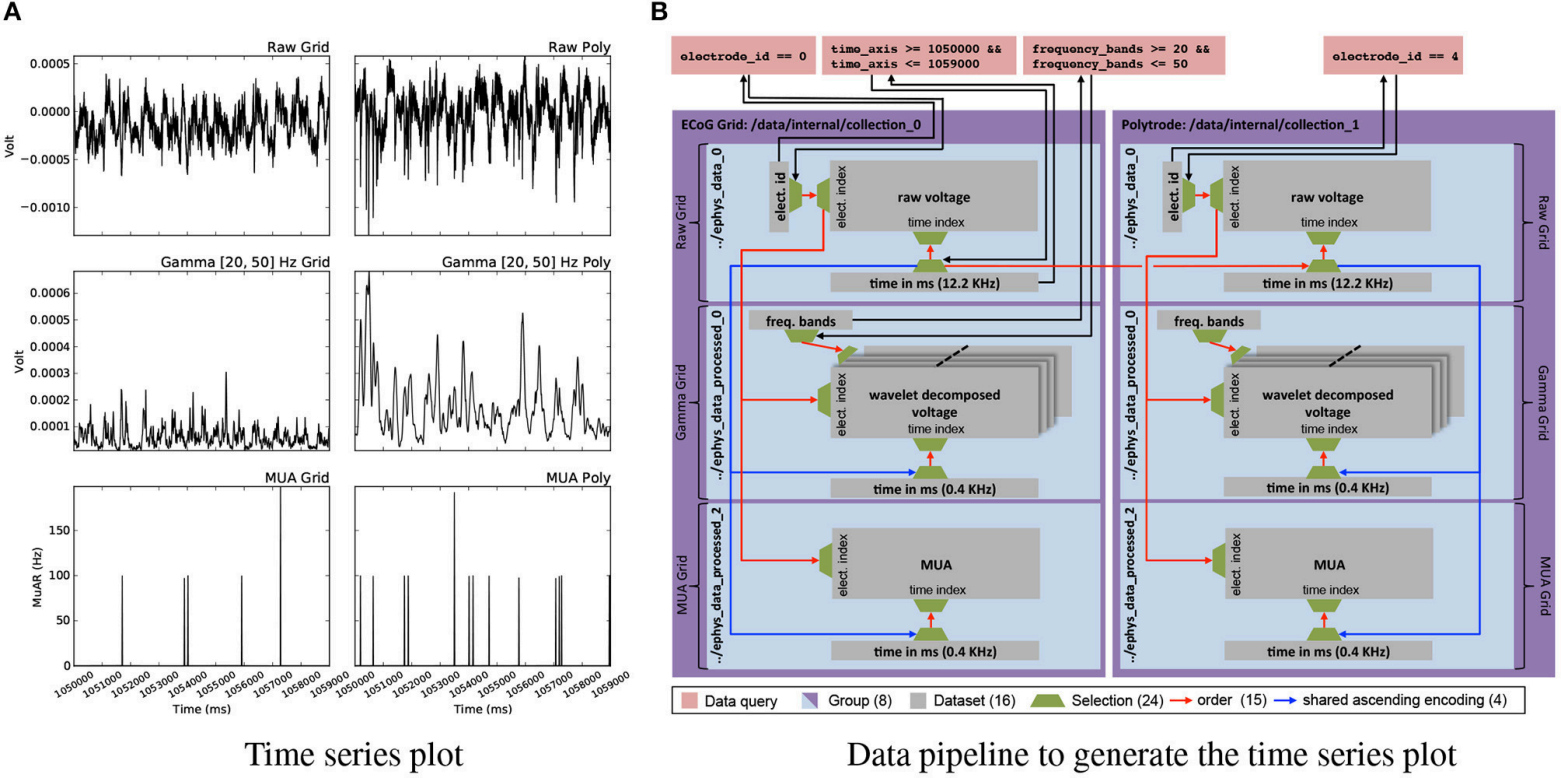
**(A)** Plot of electrode 0 of the ECoG grid data (left) and electrode 4 of the polytrode data for *time* = (1050000, 1059000)*ms* for the raw voltage recorded at 12.2*KHz* (top) and the processed gamma (middle) and multi-unit activity (MUA) (bottom) data. **(B)** Graph illustrating the construction of the plot shown in **(A)**. While the plot may appear simple, the task of simultaneously visualizing data from different modalities and processing stages requires a complex network of datasets, selections, and relationships for mapping selections to enable the reliable and consistent extraction of matching data across datasets.

**Figure 8:**
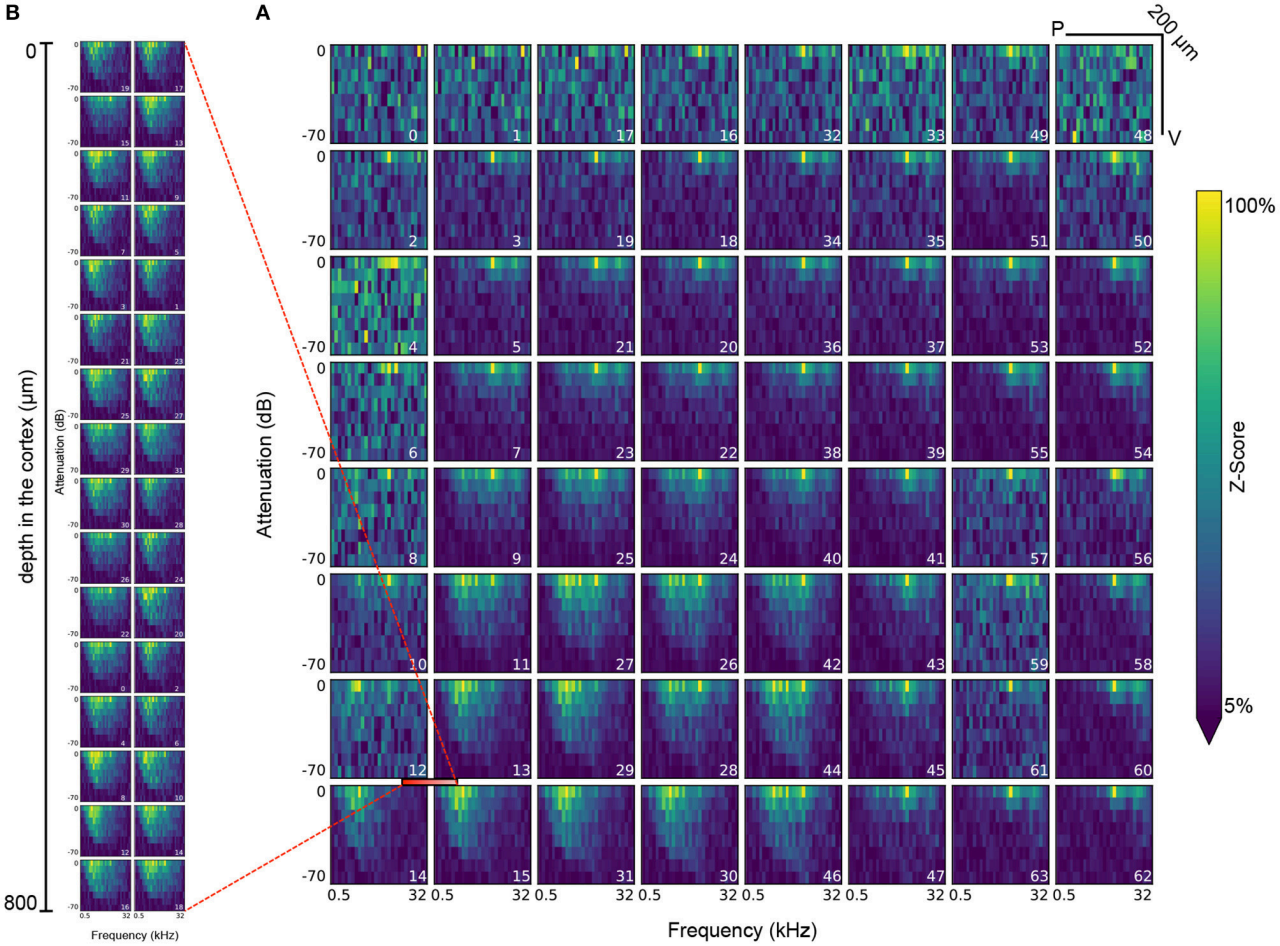
**Visualization of the response of all electrodes in the ECoG grid (A) and the polytrode (B) to tone stimuli at a frequency of 0.5 to 32 KHz and an attenuation of 0 to −70*dB***. The polytrode was inserted at the intersection of electrodes 12, 13, 14, and 15 in the μECoG grid (demarcated with red bar). Each plot represents a single electrode, while the index of the electrode as defined by the layout of the recording device is indicated in the bottom right of each plot. Each bin in a plot represents the mean of the z-scored response in the 25–50 Hz frequency band after each onset of a tone stimulus of the corresponding amplitude and frequency. Each stimulus is presented 20 times for 300*ms* each throughout the experiment.

Analytics involving data from multiple modalities and processing stages depend on the ability to map complex queries and resulting selections between datasets. Figure 7A shows a deceivingly simple example visualization of six plots showing raw voltages from an electrode on the μECoG grid and one on the polytrode, and two corresponding processed results for each. The curves show the same time range and the same electrode for the corresponding device. As Figure 7B illustrates, construction of this visualization depends on a complex network of datasets and relationships. Using relationship attributes allows us to easily store, discover, and retrieve relationships as well as search for relationships via RelationshipAttribute.find_relationships(source,target). Using relationships, we can then conveniently and reliably map selections between two datasets simply by applying the selection via array slicing to the corresponding RelationshipAttribute object and subsequently applying the mapped selection to the target dataset. The generation of the plots shown in Figures 6A,B, 7A, 8 is documented in the following Jupyter code notebook http://tinyurl.com/zq6uuja.

Figure 8 shows another example of a common analysis for the measured neural response to audio stimuli of varying frequency (0.5 to 32 KHz) and attenuation (0 to −70 dB) for all electrodes of the μECoG grid and the polytrode. This kind of analysis requires a series of datasets describing stimulus onsets, amplitudes, and frequencies and electrode layouts and responses. Identification of the relevant datasets and reliable and consistent mapping of selections for retrieval of corresponding data values then relies on a series of relationship attributes between the datasets. This plot suggests that the stimulus response recorded via the μECoG grid on the cortical surface is consistent with the response recorded by the polytrode from inside the cortex.

The formal modeling of data relationships is a powerful tool that allows us to more easily design reusable, complex multi-modal data analytics while drastically reducing the risk for errors. The ability to discover and use relationships for mapping between data also facilitates data reuse and sharing by providing critical information about dependencies between data objects and empowering users not familiar with the data to quickly and reliably create complex data analytics.

## 5. Conclusions and future work

Neuroscience is facing an incredible data challenge; novel experimental technologies generate increasingly large volumes of data (often 100s of GB to TBs) at ever faster rates while large varieties of data from different acquisition modalities are being combined to enable the integrated study of different aspects of experiments (e.g., via electrical and optical physiology, fMRI, MSI, electron and light microscopy, video and audio recording, among many others). Efficient and easy-to-use data standards are a critical foundation to solving this challenge by enabling efficient storage, management, sharing, and analysis of complex neuroscience data. Standardizing neuroscience data is as much about defining common schema and ontologies for organizing and communicating data as it is about defining basic storage layouts for specific data types. Arguably, the focus of a neuroscience-oriented standard should be on addressing the application-centric needs of organizing scientific data and metadata, rather than on reinventing file storage and format methods. For the development of BRAINformat we have used HDF5 as the basic storage format, because it already satisfies a broad range of the more basic requirements.

The complexity and variety of experiments and the diversity of data types and acquisition modalities used in neuroscience make the creation of a general, all-encompassing standard a daunting—if not futile—task. We have introduced the concept of managed objects (and managed types), which—in combination with an easy-to-use, formal format-specification document standard and API—enables us to divide and conquer the standardization problem in a modular and extensible fashion. Format components specified using these concepts can be easily reused and extended, verified for format-compliance, and stored in a self-contained and modular fashion. The format specification and managed object APIs are not specific to neuroscience, but define application-independent design concepts that enable us to efficiently create application-oriented format modules. We have demonstrated the application of these concepts to develop an extensible standard for electrophysiology data that is portable, scalable, extensible, self-describing, and that supports self-contained (single-file) and modular (multiple-linked-files) storage.

We have also introduced the novel concept of relationship attributes for modeling and use of structural and semantic relationships between data objects, including advanced index map relationships based on the notion of relationship chains. Modeling data relationships enables the structured use and analysis of related and multi-modal data, facilitates discovery, is central to provenance, and avoids potentially critical use errors. We have demonstrated the use of relationships attributes to facilitate the reliable and reusable implementation of multi-modal data analyses. Although these features are available through an API, the data stored in the format is fully specified and human readable, so that domain scientists can access the it even without our API.

We are actively working with the Bouchard lab and Denes lab at Lawrence Berkeley National Laboratory and the Chang lab at the University of California, San Francisco on the development and evaluation of data standards for neurophysiology data (the PIs and members of all three labs are also authors on this manuscript). Members of the BRAINformat team are also engaged with the Kavli Institute for Fundamental Neuroscience, San Francisco and have contributed to Neurodata Without Borders (NWB). We have demonstrated the application of BRAINformat to electrophysiology experiments by the Bouchard lab recording from the primary auditory cortex (A1) of anesthetized rats (Section 4) as well as electrocorticography (ECoG) data collected from neurosurgical patients during speech production by the Chang lab (Supplement 3). These data share many requirements with most physiology data collected by the community: storage of signals recordings over time across multiple spatially distributed sensors with potentially heterogenous geometries, complex and multi-tiered task descriptions, *post-hoc* processing of raw data to extract the signal of interest, the association of physiology with multi-modal data streams collected simultaneously by other devices, the linkage of data associated with the same task or stimulus across multiple sessions, and the necessity to store rich metadata to make sense of it all. While we have focused so far on the application of BRAINformat to electrophysiology data, a central goal of the modular, managed-object-based design has always been to facilitate extension and reuse of existing modules and creation of new format modules for integration of new information and data types in support of extended and new application use cases.

The novel concepts and capabilities introduced by the BRAINformat standardization framework fill important gaps in the portfolio of available tools for creating advanced standards for modern scientific data. Beyond just the design of a specific new format, a primary goal of our work is to provide the community with effective methods and tools to design and explore new formats. These concepts and tools provide a foundation to facilitate and inform efforts aimed at defining community-wide standards, such as the NWB (Teeters et al., 2015) initiative. In large part due to the contributions of members of a number of format development teams, including BRAINformat, NWB has adopted the use of formal, JSON-based format specification documents and a module-based format design similar to the format specification methods and managed-object-based design we have introduced here. The BRAINformat library is open source, has detailed developer documentation and user tutorials, and is freely available at: https://bitbucket.org/oruebel/brainformat.

In our future work we plan to extend BRAINformat via advanced support for metadata ontology and data type specification capabilities and efficient metadata search and data annotation modules. We will develop capabilities to enable linking and interaction with external data stored in third-party formats (e.g., movies or images) and will develop additional data modules needed to provide a broader coverage of use cases in neuroscience research. Future work on making metadata machine readable, as well as human readable, is important to accelerate the analysis of diverse data sets en masse.

## Author contributions

OR is the main developer of the presented software. MD contributed to the preparation and analysis of the rat data and design of the use case. P is the compute lead for the project. PD is the applications and engineering lead for the project. DC has contributed to the evaluation of the software. EC is the application lead from UCSF and has provided data. KB has contributed to the design and evaluation of the software and has provided data.

### Conflict of interest statement

The authors declare that the research was conducted in the absence of any commercial or financial relationships that could be construed as a potential conflict of interest.
