# Supplementary material for: Methods for Specifying Scientific Data Standards and Modeling Relationships with Applications to Neuroscience
Source: Front Neuroinform. 2016 Nov 4;10:48. doi: 10.3389/fninf.2016.00048 (PMC5095137; doi:10.3389/fninf.2016.00048)
Supplement: Supplementary file 1 [file DataSheet1.PDF]

Supplementary Material: Standardizing Neuroscience Data Formats and Modeling Data Relationships

Oliver Rübel*, Prabhat, Peter Denes, David Conant, Edward Chang, and Kristofer Bouchard

*Correspondence:
Author Name: Oliver Rübel
oruebell@lbl.gov

CONTENTS

- Supplement 1: Format Specification, License, Copyright **2 – 53**
  1. Specifying File Modules 2 – 8
  2. Full Specification for $BrainDataFile$ 9 – 40
  3. Specification Document for $brain.dataformat.brainformat$ 41 – 52
  4. License & Copyright 53
- Supplement 2: `BrainDataEPhys` Auto-Expand-Mode Example **54**
- Supplement 3: Application to electrocorticography (ECoG) during speech production **55**

SUPPLEMENT 1: FORMAT SPECIFICATION, LICENSE, COPYRIGHT

1 Specifying File Modules

Managed file modules are specified via Python dictionaries (which may be serialized to JSON). Helper datastructures are provided as part of the *brain.dataformat.spec* module. Below we define the basic structure of format specification dictionaries, but we highly recommend the use of the *brain.dataformat.spec* helper classes to ease the definition of specifications and ensure compliance of the generated documents with the document standard.

1.1 Group Specification

The group specification must contain the following keys/values:

- **datasets** : Dictionary of dataset specifications (see below) describing the datasets contained in the group. Note, these are datasets that are managed directly by this managed group. Datasets with a dedicated manager type as part of the file format are specified via the **managed_objects** key.
- **groups** : Dictionary of group specification describing the groups contained in this group. Note, these are groups that are managed directly by this managed group. Groups with a dedicated manager type as part of the file format API are specified via the **managed_objects** key.
- **managed_objects** : List of managed object specification describing additional managed datasets or groups contained in this group.
- **attributes** : List of attribute specifications. These are attributes associated with the group object directly.
- **group** : The name of the group. May be None in case the group does not have a fixed name but multiple instances of the managed group object may exist, in which case the **prefix** key should be set. In general, only one of **group** or **prefix** should be set but not both.
- **prefix** : String indicating the prefix to be used for the group name. The prefix is used in case that multiple instances of this managed object are allowed.
- **optional** : Boolean indicating whether the group is optional or mandatory.
- **description** : String describing the purpose of this managed object type. Stored in the brainformat_description attribute used to help new users with the interpretation of the format.
- **relationships** : Optional list of relationship specifications describing relationships of this group to other objects. The key may be omitted if no relationships are specified.

Example:

```
{'datasets': {'ecog_data': {'dataset': 'raw_data',              # dataset key is mandatory may be No
                            'prefix': None,         # prefix key is mandatory may be None
                            'optional': False,      # optional key is mandatory
                            # dimensions key is optional. If specified we assume that the number of
                            # dimensions is fixed and that a scale is defined for all dimensions,
                            # even if it is empty
                            # NOTE: if multiple scales are defined for the same axis, then the name
                            #       for those axis must be the same, while the unit key may differ
                            #       between scales for the same dimension
                            'dimensions': [{'name': 'space',      # Mandatory
                                            'unit': 'id',         # Mandatory
                                            'optional': False,    # Mandatory
                                            'dataset': 'electrode_id',  # Mandatory. Set to None to
                                            'axis': 0,
                                            'description': 'description': 'Id of the recording elec
                                           {'name': 'time',
                                            'unit': 'ns',
                                            'optional': False,
```

```
                                            'dataset': 'time_axis',
                                            'axis': 1,
                                            'description': 'Sample time in ns'}],  # use empty dict
                            'description': 'Dataset with the ECoG recordings data',  # Mandatory
                            'attributes': [{'attribute': 'unit',    # Mandatory but may be empty
                                            'value': 'Volt', # Mandatory may be None to indicate us
                                            'prefix': None,         # Mandatory
                                            'opional': False}]},    # Mandatory
              'sampling_rate': {'dataset': 'sampling_rate',
                                'prefix': None,
                                'optional': False,
                                'attributes': [{'attribute': 'unit',
                                                'value': 'KHz',
                                                'prefix': None,
                                                'optional': False}],
                                'description': 'Sampling rate in KHz'},
              'layout': {'dataset': 'layout',
                         'prefix': None,
                         'optional': True,
                         'dimensions': [],
                         'attributes': [],
                         'description': 'The physical layout of the electrodes.'}},
 'groups': {},           # Mandatory
 'managed_objects': [],  # e.g., {'format_type': 'BrainDataECoG', 'optional': True}
 'attributes': [],  # e.g, [{'attribute': 'unit', 'value': KHz, 'prefix': None, 'optional': False}]
 'group': None,          # Mandatory (use 'dataset' in case of a managed dataset
 'prefix': "ecog_data_",
 'optional': False,
 'description': 'Managed group for storage of raw ECoG recordings.'}
```

1.2 File Specification

The specification of managed files is very similar to the specification of group objects with the following key differences:

- **file_prefix** : Required additional entry describing the name prefix for filenames. May be set to *None* indicating that arbitrary filenames may be used.
- **file_extension** : Required file extension. May be set to *None* to indicate that arbitrary file may be used extensions.
- **group**, **prefix** : The behavior of these keys is identical to groups only that they are used to determine the name of external links to the files root group. In contrast to group specification, both *group* and *prefix* are allowed to be simultaneously set to None, indicating that no external links should be generated to the file. This is, e.g,the for *brain.dataformat.base.ManagedObjectFile* which is a pure container object with the intend that we should only link to specific object within the container but not the container file itself.

```
{'datasets': {},
 'groups': {},
 'managed_objects': [{'format_type': 'BrainDataData', 'optional': False},
                     {'format_type': 'BrainDataDescriptors', 'optional': False}],
 'attributes': [],
 'group': None,
 'prefix': "entry_",
 'file_prefix': None,
 'file_extension': '.h5',
```

```
 'optional': False,
 'description': 'Managed BRAIN file.'}
```

1.3 Managed Objects Specification

The specification of managed objects consists of the following keys/values:

- **format_type** : String indicating the type of the managed object, e.g., **BrainDataECoG**. Use **ManagedObject** as format type, to indicate that any type of managed object may be part of the current group or file.
- **optional**: Boolean indicating whether the managed object optional or mandatory. This overwrites the **optional** key of the format specification of the specification of the managed object.

Example managed object specification:

```
{'format_type': 'BrainDataECoG', 'optional': True}
```

1.4 Attribute Specification

The specification of attributes consists of the following keys/values:

- **attribute** : Fixed name for the attribute. May be None in case that a **prefix** is specified allowing multiple instances of the attribute for the same object.
- **value** : Value of the attribute. May be None in case the value for the attribute is not fixed by user-defined.
- **prefix** : Prefix for the attribute. The prefix is automatically appended by a number so that multiple instances of the attribute are possible.
- **optional**: Boolean indicating whether the managed object is optional or mandatory.
- **description**: Optional string describing the attribute in a human-readable form. The description is optional for attributes, as it cannot be saved to file as part of the attribute itself (attributes cannot have additional attributes) but only as part of the larger spec of the object the attribute is applied to

Example attribute specification:

```
{'attribute': 'unit',     # Mandatory but may be None is 'prefix' is set
 'value': 'Volt',         # Mandatory may be None to indicate that the value user defined (rather th
 'prefix': None,          # Mandatory may be None if 'attribute' is set
 'optional': False}    # Mandatory boolean.
```

1.5 Dataset Specification

The specification of datasets consists of the following keys/values:

- **dataset** : Fixed name for the dataset. May be None in case that **prefix** is specified to indicate that multiple numbered instances of the dataset type are allowed.
- **prefix** : String indicating the prefix to be used for the datset name. The prefix is used in case that multiple instances of this dataset object are allowed.
- **dimensions** : List of dimension scale specification. The **dimensions** key is optional. If specified we assume that the number of dimensions is fixed and that a scale is defined for all dimensions, even if it is empty. NOTE: if multiple scales are defined for the same axis, then the name for those axis must be the same, while the unit key may differ between scales for the same dimension

- **dimensions_fixed** Boolean indicating whether the dataset must have exactly the number of dimensions specified by dimensions. If False, then the dataset is allowed to have additional dimensions not specified in *dimensions*. This parameter is optional. If the parameter is missing and *dimensions* are specified then it is implicitly assumed to be set to True. If the parameter is missing and *dimensions* is empty then the parameter is assumed to be implicitly False.
- **description** : String describing the purpose of this managed object type. Stored in the brainformat_description attribute used to help new users with the interpretation of the format.
- **attributes** : List of attribute specifications. These are attributes associated with the group object directly.
- **optional**: Boolean indicating whether the object is optional or mandatory.
- **primary** : Boolean indicating whether the dataset is a primary data source for analysis. This attribute is optional and is assumed to be False if missing. Marking primary data sources is useful when using data files as part of a third-party visualization and analysis tools and allows third-party tools to discover which datasets are the primary sources.
- **relationships** : Optional list of relationship specifications describing relationships of this dataset to other objects. The key may be omitted if no relationships are specified.

Example dataset specification:

```
{'dataset': 'raw_data',
 'prefix': None,
 'optional': False,
 'primary': True
 'dimensions': [{'name': 'space',
                 'unit': 'id',
                 'optional': False,
                 'dataset': 'electrode_id',
                 'axis': 0},
                {'name': 'time',
                 'unit': 'ns',
                 'optional': False,
                 'dataset': 'time_axis',
                 'axis': 1}],
 'description': 'Dataset with the ECoG recordings data',
 'attributes': [{'attribute': 'unit',
                 'value': 'Volt',
                 'prefix': None,
                 'opional': False}]}
```

1.6 Dimension Scales Specification

Dimension scale specifications are an optional part of dataset specifications and are only allowed there. Dimension scales describe the name/type of a particular dimension of dataset. The specification of dimension scales consist of the following keys/values:

- **name** : The name of the dimensions scale. NOTE: if multiple dimensions scales are associated with the same axis of a dataset, then their **name** must be identical while their **unit** keys may differ. If a dimension is required but does not have a dimension scale, then set the name to None. In this case unit and dataset should be None as well.
- **unit** : The units in which the dimension is expressed. May be None in case that only a name for the dimensions should be specified but no actual dimension scale. NOTE: if a dataset is specified then unit must be set as well as this is used to address the dataset.
- **optional**: Boolean indicating whether the object is optional or mandatory.

- **dataset** : The HDF5 dataset with the values for the dimension scale. May be None, in case that no actual axis scale should be specified, but rather only the dimensions should be labeled, but no actual dimensions scale should be created. In advanced cases, this may also be a complete Dataset Specification (rather than just a name).
- **axis** : Unsigned Integer indicating the axis/dimension the dimension scale is associated with. (Mandatory)
- **description**: Description of the dimensions scale. The description is associated with the dataset as the format description attribute (i.e., if the dataset is not set to None). (Mandatory)
- **relationships** : Optional list of relationship specifications describing relationships of the dataset associated with the dimensions scale to other objects. The key may be omitted if no relationships are specified. If relationships are specified, then the **dataset** key must be set.

```
{'name': 'space',
 'unit': 'id',
 'optional': False,
 'dataset': 'electrode_id',
 'axis': 0,
 'description': 'Id of the recording electrode'}
```

1.7 Relationship Specification

Relationships describe semantic links between file objects (usually datasets or groups). They are an optional part of Dataset and Group specifications. Relationship may also be defined as part of Dimensions Scale specifications if a dataset is associated with the scale.

In practice, many relationships are dynamic (i.e., only known once the data is generated), however, some relationships can already be described in the specification of the file format itself. Such static relationships often describe basic details of data structures, e.g, one array storing indices into another array etc.. The specification of basic relationships consists of the following keys/values:

- **attribute**: The name of the attribute used to store the relationship. May be None if *prefix* is sperificed. One of *prefix* or *attribute* must be set.
- **prefix**: Prefix of attributes used to store this type of relationship. May be None if *attribute* is specified. One of *prefix* or *attribute* must be set.
- **traget**: Dictionary specifying the target object of the relationship (e.g, the dataset the relationship points to). The target dictionary consists of the following keys/values:
  - **filename** File where the target is located. Set to None in case that the target is located in the same file as the source.
  - **global_path** Typically we try to define Managed Objects in a self-contained fashion, i.e., all data related to object should be available in the same managed objects. However, in some cases it is useful to store repeatably-used and shared data in central locations. The global key allows us to specify the location of global targets that we want to point to. The *dataset*, *group*, *prefix* keys are still honored, i.e, the global key only gives us the base location where the object is located.
    - *<path>* The global key may be an absolute path, in which case the path will start with */*. This form of description is often used when a user adds custom relationships to the file.
    - *<GlobalTargetClass>:<GlobalKey>* *GlobalTargetClass* indicates the name of the dictionary (i.e, namespace) for the global targets and the *GlobalKey* is the name of the particular global target, where the value associated with the key is expected to be the absolute path within the file to the global target. This strategy is mainly useful when specifying static relationships and assumes that a dictionary of GLOBAL_PATH is available for the format

so that these paths can be resolved to absolute paths when creating the relationships. I.e., within a file, *global_paths* should always be an absolute <*path*>, but within a specification of a format this strategy may be used to avoid the inclusing of absolute path in the specification of indiviual Managed Objects and to allow global paths to be specified in a central location that the API must be aware of.

  * *None* indicating that we are indexing a local structure within the parent group of the source object

- **dataset** The name of the dataset the relationship points to. This is usually a relative name within the current specification (i.e., the parent object that contains the object with the relationship). May be None if *group* or *prefix* are defined. *dataset* must be None if *group* or *prefix* are set.
- **group** The name of the group the relationship points to. This is usually a relative name within the current specification (i.e., the parent object that contains the object with the relationship). May be None if *dataset* or *prefix* are defined. Must be None if *dataset* is set. May be used in combindation with *prefix* to point to the parent location where the prefix's are located.
- **prefix** The name prefix of the dataset or group the relationship points to . This is usually a relative name within the current specification (i.e., the parent object that contains the object with the relationship). May be None if *group* or *dataset* are defined. May be used in combination with *group*.
- **axis** The index (or name) of the axis we point to (if the relationship points to a particular dataset) or None. Must be None if the relationship points to a group. May be a list of axis indicies if the relationship encompasses multiple axis.

• **axis**: The axis of the source object the relationships refers to (if the source is a dataset). May be a list of axis indicies if the relationship encompasses multiple axis. Use None if the relationship does not refer to a particular axis but rather the source object as a whole. (Must be None if the relationship refers to a group). In the case of *indexes* relationships, axis may be used to identify the dimension that defines the indicies (e.g, if we index a 2D dataset then we may have a 20x2 dataset containing 20 two-dimensional indicies, where the second axis defines the indicies). Also, in *indexes* relationships, this may also be a dict to encode the {'INDEXING_AXIS':<value>, 'STACK_AXIS':<value>}, i.e., the axis used for indexing and the axis used for stacking to describe 1 to many indexing.

• **relationship_type**: String indicating the type of the relationship. Currently supported relationship types include:

- *indexes*: The *source* dataset contains indices into the target dataset. These are often integer indices, however, e.g, if the relationship points to a group, then the source dataset may also contain strings selecting the objects stored in the group. The source of such a relationship, however, should always be a dataset. In the case of multi-dimensions indicies it is useful to specify *axis* for the *source* to indicate along which dimensions (typically 0 or the last dimensions) indices are stored.
- *indexes_values*: The *source* selects certain parts of the *target* based on the values (or keys in case of group(s)) in the *target*. Specification of axis for the *target* usually does not make sense for this type of relationship. The *indexes_values* relationship implies that the datasets use a *shared_encoding* (see next bullet) and is effectively a special type of *shared_encoding* relationship that beyond the encoding describes that the *source* is selecting data in the *target* based on value.
- *shared_encoding*: The target and source dataset contain values with the same encoding, i.e., values in the two datasets can be directly compared. The specification of a target axis usually does not make sense for this type of relationship.
- *shared_ascending_encoding* : Same as *shared_encoding* but the source and target datasets are expected to be sorted by value (e.g., in the case of time)

  - *order*: The ordering of objects matches between the datasets along the given axes. This relationship type in practice makes mainly sense between datasets as no explicit order is defined for objects inside a group (however a user may impose a particular order in their formats if desired).
  - *equivalent*: In addition to order, this relationship type expresses that the source and target object encode the same data (even if they might store different values). E.g., a token may be encoded by its name or by an integer index. This relationship also implies that the source and target contain the same number of values ordered in the same fashion.
  - *user*: Arbitrary user-defined relationship. Use this type to describe arbitrary relationships between objects. E.g, two datasets may have some semantic relationship that a user may want to document. Additional data to describe this relationship may be stored in the *properties* of the relationship.
- **optional**: Boolean indicating whether the object is optional or mandatory
- **description**: Text describing the relationship in a human-readable form
- **properties**: Optional (JSON serializable) dictionary with additional user properties.

NOTE: In the case of relationships we assume that both the *source* and *target* have already been specified, i.e., we can here only refer to the data by name and not via Dataset or Group specifications. **NOTE:** Relationships may generally only be defined for Datasets and Groups (including Datasets of DimensionScales) but not for Attributes.

```
{'attribute':'region_encoding',          # The name of the relationship
 'prefix': None,                         # Attribute is set
 'target': {'global': None,              # None if this is a local relationship or <GlobalTargetCla
            'dataset': 'anatomy_id',     # The dataset we link to
            'group': None,               # The group we link to
            'prefix': None,              # The prefix of the object(s) we link to
            'prefix_index': None,        # The index of the prefix object we link to or None if we
            'axis': None},               # The axis of the dataset we link to
 'axis': None,                           # The axis that has the relationship
 'type': 'equivalent',                   # The type of the relationship.
 'optional': False,                      # Is the relationship optional
 'description':' Region encoded as id',  # Text description of the relationship
 'properties': None                      # Optional dict with user properties
}
```

1.8 Format Document Specification

A format document is a dictionary with all managed types and their specification. The keys of the document are strings with the names of the managed types/classes and the values are the specifications of the managed object type.

2 Full Specification for BrainDataFile (JSON)

Since the format is directly specified by the ManagedObjects we can easily construct the full specification for the full file format (e.g, for *BrainDataFile*) as follows:

```
import time
from brain.dataformat.brainformat import BrainDataFile
from brain.dataformat.spec import *

format_spec = BrainDataFile.get_format_specification_recursive()
file_spec = BaseSpec.from_dict(format_spec)

print '**' + str(time.ctime(time.time())) + '**'
print file_spec.to_json(pretty=True)
```

An example output from the above code-example is given next. Similarly we can compute the specification of any managed sub-object of a file.

```
**Mon Sep 19 18:08:39 2016**
{
    "attributes": [
        {
            "attribute": "format_type",
            "optional": false,
            "prefix": null,
            "value": "BrainDataFile"
        },
        {
            "attribute": "format_description",
            "optional": false,
            "prefix": null,
            "value": "Managed BRAIN file."
        },
        {
            "attribute": "object_id",
            "optional": true,
            "prefix": null,
            "value": null
        },
        {
            "attribute": "format_specification",
            "optional": false,
            "prefix": null,
            "value": null
        }
    ],
    "datasets": {},
    "description": "Managed BRAIN file.",
    "file_extension": ".h5",
    "file_prefix": null,
    "group": null,
    "groups": {
        "data": {
            "attributes": [
                {
                    "attribute": "format_type",
                    "optional": false,
                    "prefix": null,
                    "value": "BrainDataData"
                },
                {
                    "attribute": "format_description",
                    "optional": false,
                    "prefix": null,
                    "value": "Managed group for storage of brain data (internal and external)."
                },
                {
                    "attribute": "object_id",
                    "optional": true,
                    "prefix": null,
                    "value": null
                },
                {
                    "attribute": "format_specification",
                    "optional": false,
                    "prefix": null,
                    "value": null
                }
            ],
            "datasets": {},
            "description": "Managed group for storage of brain data (internal and external).",
            "group": "data",
            "groups": {
                "external": {
                    "attributes": [
                        {
                            "attribute": "format_type",
                            "optional": false,
                            "prefix": null,
                            "value": "BrainDataExternalData"
                        },
```

```
                        {
                            "attribute": "format_description",
                            "optional": false,
                            "prefix": null,
                            "value": "Managed group for storage of external data related to the internal brain data."
                        },
                        {
                            "attribute": "object_id",
                            "optional": true,
                            "prefix": null,
                            "value": null
                        },
                        {
                            "attribute": "format_specification",
                            "optional": false,
                            "prefix": null,
                            "value": null
                        }
                    ],
                    "datasets": {},
                    "description": "Managed group for storage of external data related to the internal brain data.",
                    "group": "external",
                    "groups": {
                        "stimulus_": {
                            "attributes": [
                                {
                                    "attribute": "format_type",
                                    "optional": false,
                                    "prefix": null,
                                    "value": "BrainDataStimulus"
                                },
                                {
                                    "attribute": "format_description",
                                    "optional": false,
                                    "prefix": null,
                                    "value": "Group stroing metadata about a stimulus"
                                },
                                {
                                    "attribute": "object_id",
                                    "optional": true,
                                    "prefix": null,
                                    "value": null
                                },
                                {
                                    "attribute": "format_specification",
                                    "optional": false,
                                    "prefix": null,
                                    "value": null
                                }
                            ],
                            "datasets": {
                                "_": {
                                    "attributes": [
                                        {
                                            "attribute": "unit",
                                            "description": "Attribute describing the units of the metadata",
                                            "optional": true,
                                            "prefix": null,
                                            "value": null
                                        },
                                        {
                                            "attribute": "ontology",
                                            "description": "Attribute describing the ontology used for the metadata",
                                            "optional": true,
                                            "prefix": null,
                                            "value": null
                                        },
                                        {
                                            "attribute": "user_description",
                                            "description": "Attribute describing the purpose of the dataset",
```

```
                                        "optional": true,
                                        "prefix": null,
                                        "value": null
                                    },
                                    {
                                        "attribute": "format_type",
                                        "optional": false,
                                        "prefix": null,
                                        "value": "BrainDataMetadataDataset"
                                    },
                                    {
                                        "attribute": "format_description",
                                        "optional": false,
                                        "prefix": null,
                                        "value": "Metadata storage dataset"
                                    },
                                    {
                                        "attribute": "object_id",
                                        "optional": true,
                                        "prefix": null,
                                        "value": null
                                    },
                                    {
                                        "attribute": "format_specification",
                                        "optional": false,
                                        "prefix": null,
                                        "value": null
                                    }
                                ],
                                "dataset": null,
                                "description": "Metadata storage dataset",
                                "dimensions": [],
                                "optional": true,
                                "prefix": null,
                                "relationships": []
                            },
                            "type": {
                                "attributes": [],
                                "dataset": "type",
                                "description": "The type of stimulus",
                                "dimensions": [],
                                "optional": false,
                                "prefix": null,
                                "relationships": []
                            }
                        },
                        "description": "Group stroing metadata about a stimulus",
                        "group": null,
                        "groups": {},
                        "managed_objects": [],
                        "optional": true,
                        "prefix": "stimulus_",
                        "relationships": []
                    }
                },
                "managed_objects": [],
                "optional": false,
                "prefix": null,
                "relationships": []
            },
            "internal": {
                "attributes": [
                    {
                        "attribute": "format_type",
                        "optional": false,
                        "prefix": null,
                        "value": "BrainDataInternalData"
                    },
                    {
                        "attribute": "format_description",
                        "optional": false,
                        "prefix": null,
                        "value": "Managed group for storage of a collection of internal brain data."
```

```
            },
            {
                "attribute": "object_id",
                "optional": true,
                "prefix": null,
                "value": null
            },
            {
                "attribute": "format_specification",
                "optional": false,
                "prefix": null,
                "value": null
            }
        ],
        "datasets": {},
        "description": "Managed group for storage of a collection of internal brain data.",
        "group": "internal",
        "groups": {
            "collection_": {
                "attributes": [
                    {
                        "attribute": "format_type",
                        "optional": false,
                        "prefix": null,
                        "value": "BrainDataCollection"
                    },
                    {
                        "attribute": "format_description",
                        "optional": false,
                        "prefix": null,
                        "value": "Container for storing a collection of related datasets, e.g. from a single session or modality"
                    },
                    {
                        "attribute": "object_id",
                        "optional": true,
                        "prefix": null,
                        "value": null
                    },
                    {
                        "attribute": "format_specification",
                        "optional": false,
                        "prefix": null,
                        "value": null
                    }
                ],
                "datasets": {},
                "description": "Container for storing a collection of related datasets, e.g. from a single session or modality",
                "group": null,
                "groups": {
                    "ephys_data_": {
                        "attributes": [
                            {
                                "attribute": "format_type",
                                "optional": false,
                                "prefix": null,
                                "value": "BrainDataEphys"
                            },
                            {
                                "attribute": "format_description",
                                "optional": false,
                                "prefix": null,
                                "value": "Managed group for storage of raw Ephys recordings."
                            },
                            {
                                "attribute": "object_id",
                                "optional": true,
                                "prefix": null,
                                "value": null
                            },
                            {
```

```
                        "attribute": "format_specification",
                        "optional": false,
                        "prefix": null,
                        "value": null
                    }
                ],
                "datasets": {
                    "ephys_data": {
                        "attributes": [
                            {
                                "attribute": "unit",
                                "optional": false,
                                "prefix": null,
                                "value": "Volt"
                            }
                        ],
                        "dataset": "raw_data",
                        "description": "Dataset with the Ephys recordings data",
                        "dimensions": [
                            {
                                "axis": 0,
                                "dataset": "electrode_id",
                                "description": "Id of the recording electrode",
                                "name": "space",
                                "optional": false,
                                "relationships": [],
                                "unit": "id"
                            },
                            {
                                "axis": 1,
                                "dataset": "time_axis",
                                "description": "Sample time in ms",
                                "name": "time",
                                "optional": false,
                                "relationships": [],
                                "unit": "ms"
                            },
                            {
                                "axis": 0,
                                "dataset": "anatomy_name",
                                "description": "Name of region location of the electrodes",
                                "name": "space",
                                "optional": true,
                                "relationships": [],
                                "unit": "region name"
                            },
                            {
                                "axis": 0,
                                "dataset": "anatomy_id",
                                "description": "Integer id of the region location of the electrodes",
                                "name": "space",
                                "optional": true,
                                "relationships": [],
                                "unit": "region id"
                            }
                        ],
                        "dimensions_fixed": true,
                        "optional": false,
                        "prefix": null,
                        "primary": true,
                        "relationships": []
                    },
                    "layout": {
                        "attributes": [],
                        "dataset": "layout",
                        "description": "The physical layout of the electrodes.",
                        "dimensions": [],
                        "optional": true,
                        "prefix": null,
                        "relationships": []
                    },
                    "sampling_rate": {
```

```
        "attributes": [
          {
            "attribute": "unit",
            "optional": false,
            "prefix": null,
            "value": "Hz"
          }
        ],
        "dataset": "sampling_rate",
        "description": "Sampling rate in Hz",
        "dimensions": [],
        "optional": false,
        "prefix": null,
        "relationships": []
      }
    },
    "description": "Managed group for storage of raw Ephys recordings.",
    "group": null,
    "groups": {
      "annotations_": {
        "attributes": [
          {
            "attribute": "collection_description",
            "optional": false,
            "prefix": null,
            "value": null
          },
          {
            "attribute": "format_type",
            "optional": false,
            "prefix": null,
            "value": "AnnotationDataGroup"
          },
          {
            "attribute": "format_description",
            "optional": false,
            "prefix": null,
            "value": "Managed group for storage of a collection of annotations. Multiple annotation collections may typically be associated with the same data object."
          },
          {
            "attribute": "object_id",
            "optional": true,
            "prefix": null,
            "value": null
          },
          {
            "attribute": "format_specification",
            "optional": false,
            "prefix": null,
            "value": null
          }
        ],
        "datasets": {
          "annotation_type_indexes": {
            "attributes": [],
            "dataset": "annotation_type_indexes",
            "description": "Dataset indicating for each selection the index of the annotation type used. The annotation types are given in the annotation types dataset.",
            "dimensions": [
              {
                "axis": 0,
                "dataset": null,
                "description": "Integer index into the annotation_types array indicating the type of the annotation",
                "name": "type_index",
                "optional": false,
                "relationships": [],
                "unit": null
              }
            ],
```

```
              "dimensions_fixed": true,
              "optional": false,
              "prefix": null,
              "relationships": [
                {
                  "attribute": "indexes_annotation_types",
                  "axis": 0,
                  "description": "Relationship documentation that we are storing references to annotation_types",
                  "optional": false,
                  "prefix": null,
                  "properties": null,
                  "relationship_type": "indexes",
                  "target": {
                    "axis": 0,
                    "dataset": "annotation_types",
                    "filename": null,
                    "global_path": null,
                    "group": null,
                    "prefix": null,
                    "prefix_all": null
                  }
                }
              ]
            },
            "annotation_types": {
              "attributes": [],
              "dataset": "annotation_types",
              "description": "List of all available annotation types",
              "dimensions": [
                {
                  "axis": 0,
                  "dataset": null,
                  "description": "Integer index of the type",
                  "name": "type_index",
                  "optional": false,
                  "relationships": [],
                  "unit": null
                }
              ],
              "dimensions_fixed": true,
              "optional": false,
              "prefix": null,
              "relationships": []
            },
            "data_object": {
              "attributes": [],
              "dataset": "data_object",
              "description": null,
              "dimensions": [],
              "optional": false,
              "prefix": null,
              "relationships": []
            },
            "descriptions": {
              "attributes": [],
              "dataset": "descriptions",
              "description": "Dataset with the annotation descriptions.",
              "dimensions": [
                {
                  "axis": 0,
                  "dataset": null,
                  "description": "Integer index of the annotation",
                  "name": "annotation_index",
                  "optional": false,
                  "relationships": [],
                  "unit": null
                }
              ],
              "dimensions_fixed": true,
              "optional": false,
              "prefix": null,
              "relationships": []
```

```
},
"properties": {
  "attributes": [
    {
      "attribute": "name",
      "optional": false,
      "prefix": null,
      "value": null
    }
  ],
  "dataset": null,
  "description": "Datasets with a particular property for all annotations.",
  "dimensions": [
    {
      "axis": 0,
      "dataset": null,
      "description": "Integer index of the selection",
      "name": "annotation_index",
      "optional": false,
      "relationships": [],
      "unit": null
    }
  ],
  "dimensions_fixed": false,
  "optional": true,
  "prefix": "property_",
  "relationships": []
},
"selection_indexes": {
  "attributes": [],
  "dataset": "selection_indexes",
  "description": "Dataset indicating for each axis the index of the selection applied to the given axis. -1 indicates that no selection is applied along that axis. The axis index ranges from -1 to n where -1 indicated global selection and n is the number of axes.",
  "dimensions": [
    {
      "axis": 0,
      "dataset": null,
      "description": "Integer index of the annotation",
      "name": "annotation_index",
      "optional": false,
      "relationships": [],
      "unit": null
    },
    {
      "axis": 1,
      "dataset": "axis_index",
      "description": "Integer index of the axis",
      "name": "axis_index",
      "optional": false,
      "relationships": [
        {
          "attribute": "select_axis",
          "axis": null,
          "description": "Relationship documentation that each column of the selection_indexes dataset refers to a different selections_axis_ dataset",
          "optional": false,
          "prefix": null,
          "properties": null,
          "relationship_type": "order",
          "target": {
            "axis": null,
            "dataset": null,
            "filename": null,
            "global_path": null,
            "group": null,
            "prefix": "selections_axis_",
            "prefix_all": null
          }
        }
      ],
```

```
                            "unit": "index"
                        }
                    ],
                    "dimensions_fixed": true,
                    "optional": false,
                    "prefix": null,
                    "relationships": []
                },
                "selections": {
                    "attributes": [
                        {
                            "attribute": "axis",
                            "optional": false,
                            "prefix": null,
                            "value": null
                        }
                    ],
                    "dataset": null,
                    "description": "Datasets with all selections for the indicated axis. Axis -1 indicates a global selection across all axes. One dataset per axis and one for global selection (-1) is mandatory.",
                    "dimensions": [
                        {
                            "axis": 0,
                            "dataset": null,
                            "description": "Integer index of the selection",
                            "name": "selection_index",
                            "optional": false,
                            "relationships": [],
                            "unit": null
                        }
                    ],
                    "dimensions_fixed": false,
                    "optional": false,
                    "prefix": "selections_axis_",
                    "relationships": []
                }
            },
            "description": "Managed group for storage of a collection of annotations. Multiple annotation collections may typically be associated with the same data object.",
            "group": null,
            "groups": {},
            "managed_objects": [],
            "optional": true,
            "prefix": "annotations_",
            "relationships": []
        }
    },
    "managed_objects": [],
    "optional": true,
    "prefix": "ephys_data_",
    "relationships": []
},
"ephys_data_processed_": {
    "attributes": [
        {
            "attribute": "format_type",
            "optional": false,
            "prefix": null,
            "value": "BrainDataEphysProcessed"
        },
        {
            "attribute": "format_description",
            "optional": false,
            "prefix": null,
            "value": "Managed group for storage of processed Ephys recordings."
        },
        {
            "attribute": "object_id",
            "optional": true,
            "prefix": null,
```

```
            "value": null
        },
        {
            "attribute": "format_specification",
            "optional": false,
            "prefix": null,
            "value": null
        }
    ],
    "datasets": {
        "ephys_data": {
            "attributes": [
                {
                    "attribute": "unit",
                    "optional": false,
                    "prefix": null,
                    "value": null
                },
                {
                    "attribute": "original_name",
                    "optional": true,
                    "prefix": null,
                    "value": null
                }
            ],
            "dataset": "processed_data",
            "description": "Dataset with the Ephys recordings data",
            "dimensions": [
                {
                    "axis": 0,
                    "dataset": "spatial_id",
                    "description": "Id of the recording electrode",
                    "name": "space",
                    "optional": false,
                    "relationships": [],
                    "unit": "id"
                },
                {
                    "axis": 1,
                    "dataset": "time_axis",
                    "description": "Sample time in ms",
                    "name": "time",
                    "optional": false,
                    "relationships": [],
                    "unit": "ms"
                },
                {
                    "axis": 0,
                    "dataset": "anatomy_name",
                    "description": "Name of region location of the electrodes",
                    "name": "space",
                    "optional": true,
                    "relationships": [],
                    "unit": "region name"
                },
                {
                    "axis": 0,
                    "dataset": "anatomy_id",
                    "description": "Integer id of the region location of the electrodes",
                    "name": "space",
                    "optional": true,
                    "relationships": [],
                    "unit": "region id"
                },
                {
                    "axis": 2,
                    "dataset": "frequency_bands",
                    "description": "Frequency bands of the channels",
                    "name": "channels",
                    "optional": true,
                    "relationships": [],
                    "unit": "Hz"
```

```
      },
      {
        "axis": 2,
        "dataset": "token_id",
        "description": "Integer Id of the token type",
        "name": "channels",
        "optional": true,
        "relationships": [],
        "unit": "token id"
      },
      {
        "axis": 2,
        "dataset": "token_name",
        "description": "Name of the token type",
        "name": "channels",
        "optional": true,
        "relationships": [],
        "unit": "token name"
      }
    ],
    "dimensions_fixed": true,
    "optional": false,
    "prefix": null,
    "primary": true,
    "relationships": []
  },
  "layout": {
    "attributes": [],
    "dataset": "layout",
    "description": "The physical layout of the electrodes.",
    "dimensions": [],
    "optional": true,
    "prefix": null,
    "relationships": []
  },
  "sampling_rate": {
    "attributes": [
      {
        "attribute": "unit",
        "optional": false,
        "prefix": null,
        "value": "Hz"
      }
    ],
    "dataset": "sampling_rate",
    "description": "Sampling rate in Hz",
    "dimensions": [],
    "optional": false,
    "prefix": null,
    "relationships": []
  }
},
"description": "Managed group for storage of processed Ephys recordings.",
"group": null,
"groups": {
  "annotations_": {
    "attributes": [
      {
        "attribute": "collection_description",
        "optional": false,
        "prefix": null,
        "value": null
      },
      {
        "attribute": "format_type",
        "optional": false,
        "prefix": null,
        "value": "AnnotationDataGroup"
      },
      {
        "attribute": "format_description",
        "optional": false,
```

```
                        "prefix": null,
                        "value": "Managed group for storage of a collection of annotations. Multiple annotation collections may typically be associated with the same data object."
                    },
                    {
                        "attribute": "object_id",
                        "optional": true,
                        "prefix": null,
                        "value": null
                    },
                    {
                        "attribute": "format_specification",
                        "optional": false,
                        "prefix": null,
                        "value": null
                    }
                ],
                "datasets": {
                    "annotation_type_indexes": {
                        "attributes": [],
                        "dataset": "annotation_type_indexes",
                        "description": "Dataset indicating for each selection the index of the annotation type used. The annotation types are given in the annotation types dataset.",
                        "dimensions": [
                            {
                                "axis": 0,
                                "dataset": null,
                                "description": "Integer index into the annotation_types array indicating the type of the annotation",
                                "name": "type_index",
                                "optional": false,
                                "relationships": [],
                                "unit": null
                            }
                        ],
                        "dimensions_fixed": true,
                        "optional": false,
                        "prefix": null,
                        "relationships": [
                            {
                                "attribute": "indexes_annotation_types",
                                "axis": 0,
                                "description": "Relationship documentation that we are storing references to annotation_types",
                                "optional": false,
                                "prefix": null,
                                "properties": null,
                                "relationship_type": "indexes",
                                "target": {
                                    "axis": 0,
                                    "dataset": "annotation_types",
                                    "filename": null,
                                    "global_path": null,
                                    "group": null,
                                    "prefix": null,
                                    "prefix_all": null
                                }
                            }
                        ]
                    },
                    "annotation_types": {
                        "attributes": [],
                        "dataset": "annotation_types",
                        "description": "List of all available annotation types",
                        "dimensions": [
                            {
                                "axis": 0,
                                "dataset": null,
                                "description": "Integer index of the type",
                                "name": "type_index",
                                "optional": false,
```

```
                        "relationships": [],
                        "unit": null
                    }
                ],
                "dimensions_fixed": true,
                "optional": false,
                "prefix": null,
                "relationships": []
            },
            "data_object": {
                "attributes": [],
                "dataset": "data_object",
                "description": null,
                "dimensions": [],
                "optional": false,
                "prefix": null,
                "relationships": []
            },
            "descriptions": {
                "attributes": [],
                "dataset": "descriptions",
                "description": "Dataset with the annotation descriptions.",
                "dimensions": [
                    {
                        "axis": 0,
                        "dataset": null,
                        "description": "Integer index of the annotation",
                        "name": "annotation_index",
                        "optional": false,
                        "relationships": [],
                        "unit": null
                    }
                ],
                "dimensions_fixed": true,
                "optional": false,
                "prefix": null,
                "relationships": []
            },
            "properties": {
                "attributes": [
                    {
                        "attribute": "name",
                        "optional": false,
                        "prefix": null,
                        "value": null
                    }
                ],
                "dataset": null,
                "description": "Datasets with a particular property for all annotations.",
                "dimensions": [
                    {
                        "axis": 0,
                        "dataset": null,
                        "description": "Integer index of the selection",
                        "name": "annotation_index",
                        "optional": false,
                        "relationships": [],
                        "unit": null
                    }
                ],
                "dimensions_fixed": false,
                "optional": true,
                "prefix": "property_",
                "relationships": []
            },
            "selection_indexes": {
                "attributes": [],
                "dataset": "selection_indexes",
                "description": "Dataset indicating for each axis the index of the selection applied to the given axis. -1 indicates that no selection is applied along that axis. The axis index ranges from -1 to n where -1 indicated global selection and n is the number of axes.",
```

```
        "dimensions": [
            {
                "axis": 0,
                "dataset": null,
                "description": "Integer index of the annotation",
                "name": "annotation_index",
                "optional": false,
                "relationships": [],
                "unit": null
            },
            {
                "axis": 1,
                "dataset": "axis_index",
                "description": "Integer index of the axis",
                "name": "axis_index",
                "optional": false,
                "relationships": [
                    {
                        "attribute": "select_axis",
                        "axis": null,
                        "description": "Relationship documentation that each column of the selection_indexes dataset refers to a different selections_axis_ dataset",
                        "optional": false,
                        "prefix": null,
                        "properties": null,
                        "relationship_type": "order",
                        "target": {
                            "axis": null,
                            "dataset": null,
                            "filename": null,
                            "global_path": null,
                            "group": null,
                            "prefix": "selections_axis_",
                            "prefix_all": null
                        }
                    }
                ],
                "unit": "index"
            }
        ],
        "dimensions_fixed": true,
        "optional": false,
        "prefix": null,
        "relationships": []
    },
    "selections": {
        "attributes": [
            {
                "attribute": "axis",
                "optional": false,
                "prefix": null,
                "value": null
            }
        ],
        "dataset": null,
        "description": "Datasets with all selections for the indicated axis. Axis -1 indicates a global selection across all axes. One dataset per axis and one for global selection (-1) is mandatory.",
        "dimensions": [
            {
                "axis": 0,
                "dataset": null,
                "description": "Integer index of the selection",
                "name": "selection_index",
                "optional": false,
                "relationships": [],
                "unit": null
            }
        ],
        "dimensions_fixed": false,
        "optional": false,
        "prefix": "selections_axis_",
        "relationships": []
```

```
                        }
                    },
                    "description": "Managed group for storage of a collection of annotations. Multiple annotation collections may typically be associated with the same data object.",
                    "group": null,
                    "groups": {},
                    "managed_objects": [],
                    "optional": true,
                    "prefix": "annotations_",
                    "relationships": []
                }
            },
            "managed_objects": [],
            "optional": true,
            "prefix": "ephys_data_processed_",
            "relationships": []
        }
    },
    "managed_objects": [],
    "optional": true,
    "prefix": "collection_",
    "relationships": []
},
"ephys_data_": {
    "attributes": [
        {
            "attribute": "format_type",
            "optional": false,
            "prefix": null,
            "value": "BrainDataEphys"
        },
        {
            "attribute": "format_description",
            "optional": false,
            "prefix": null,
            "value": "Managed group for storage of raw Ephys recordings."
        },
        {
            "attribute": "object_id",
            "optional": true,
            "prefix": null,
            "value": null
        },
        {
            "attribute": "format_specification",
            "optional": false,
            "prefix": null,
            "value": null
        }
    ],
    "datasets": {
        "ephys_data": {
            "attributes": [
                {
                    "attribute": "unit",
                    "optional": false,
                    "prefix": null,
                    "value": "Volt"
                }
            ],
            "dataset": "raw_data",
            "description": "Dataset with the Ephys recordings data",
            "dimensions": [
                {
                    "axis": 0,
                    "dataset": "electrode_id",
                    "description": "Id of the recording electrode",
                    "name": "space",
                    "optional": false,
                    "relationships": [],
                    "unit": "id"
                },
```

```
                    {
                        "axis": 1,
                        "dataset": "time_axis",
                        "description": "Sample time in ms",
                        "name": "time",
                        "optional": false,
                        "relationships": [],
                        "unit": "ms"
                    },
                    {
                        "axis": 0,
                        "dataset": "anatomy_name",
                        "description": "Name of region location of the electrodes",
                        "name": "space",
                        "optional": true,
                        "relationships": [],
                        "unit": "region name"
                    },
                    {
                        "axis": 0,
                        "dataset": "anatomy_id",
                        "description": "Integer id of the region location of the electrodes",
                        "name": "space",
                        "optional": true,
                        "relationships": [],
                        "unit": "region id"
                    }
                ],
                "dimensions_fixed": true,
                "optional": false,
                "prefix": null,
                "primary": true,
                "relationships": []
            },
            "layout": {
                "attributes": [],
                "dataset": "layout",
                "description": "The physical layout of the electrodes.",
                "dimensions": [],
                "optional": true,
                "prefix": null,
                "relationships": []
            },
            "sampling_rate": {
                "attributes": [
                    {
                        "attribute": "unit",
                        "optional": false,
                        "prefix": null,
                        "value": "Hz"
                    }
                ],
                "dataset": "sampling_rate",
                "description": "Sampling rate in Hz",
                "dimensions": [],
                "optional": false,
                "prefix": null,
                "relationships": []
            }
        },
        "description": "Managed group for storage of raw Ephys recordings.",
        "group": null,
        "groups": {
            "annotations_": {
                "attributes": [
                    {
                        "attribute": "collection_description",
                        "optional": false,
                        "prefix": null,
                        "value": null
                    },
                    {
```

```
                    "attribute": "format_type",
                    "optional": false,
                    "prefix": null,
                    "value": "AnnotationDataGroup"
                },
                {
                    "attribute": "format_description",
                    "optional": false,
                    "prefix": null,
                    "value": "Managed group for storage of a collection of annotations. Multiple annotation collections may typically be associated with the same data object."
                },
                {
                    "attribute": "object_id",
                    "optional": true,
                    "prefix": null,
                    "value": null
                },
                {
                    "attribute": "format_specification",
                    "optional": false,
                    "prefix": null,
                    "value": null
                }
            ],
            "datasets": {
                "annotation_type_indexes": {
                    "attributes": [],
                    "dataset": "annotation_type_indexes",
                    "description": "Dataset indicating for each selection the index of the annotation type used. The annotation types are given in the annotation types dataset.",
                    "dimensions": [
                        {
                            "axis": 0,
                            "dataset": null,
                            "description": "Integer index into the annotation_types array indicating the type of the annotation",
                            "name": "type_index",
                            "optional": false,
                            "relationships": [],
                            "unit": null
                        }
                    ],
                    "dimensions_fixed": true,
                    "optional": false,
                    "prefix": null,
                    "relationships": [
                        {
                            "attribute": "indexes_annotation_types",
                            "axis": 0,
                            "description": "Relationship documentation that we are storing references to annotation_types",
                            "optional": false,
                            "prefix": null,
                            "properties": null,
                            "relationship_type": "indexes",
                            "target": {
                                "axis": 0,
                                "dataset": "annotation_types",
                                "filename": null,
                                "global_path": null,
                                "group": null,
                                "prefix": null,
                                "prefix_all": null
                            }
                        }
                    ]
                },
                "annotation_types": {
                    "attributes": [],
                    "dataset": "annotation_types",
                    "description": "List of all available annotation types",
```

```
            "dimensions": [
                {
                    "axis": 0,
                    "dataset": null,
                    "description": "Integer index of the type",
                    "name": "type_index",
                    "optional": false,
                    "relationships": [],
                    "unit": null
                }
            ],
            "dimensions_fixed": true,
            "optional": false,
            "prefix": null,
            "relationships": []
        },
        "data_object": {
            "attributes": [],
            "dataset": "data_object",
            "description": null,
            "dimensions": [],
            "optional": false,
            "prefix": null,
            "relationships": []
        },
        "descriptions": {
            "attributes": [],
            "dataset": "descriptions",
            "description": "Dataset with the annotation descriptions.",
            "dimensions": [
                {
                    "axis": 0,
                    "dataset": null,
                    "description": "Integer index of the annotation",
                    "name": "annotation_index",
                    "optional": false,
                    "relationships": [],
                    "unit": null
                }
            ],
            "dimensions_fixed": true,
            "optional": false,
            "prefix": null,
            "relationships": []
        },
        "properties": {
            "attributes": [
                {
                    "attribute": "name",
                    "optional": false,
                    "prefix": null,
                    "value": null
                }
            ],
            "dataset": null,
            "description": "Datasets with a particular property for all annotations.",
            "dimensions": [
                {
                    "axis": 0,
                    "dataset": null,
                    "description": "Integer index of the selection",
                    "name": "annotation_index",
                    "optional": false,
                    "relationships": [],
                    "unit": null
                }
            ],
            "dimensions_fixed": false,
            "optional": true,
            "prefix": "property_",
            "relationships": []
        },
```

```
                "selection_indexes": {
                    "attributes": [],
                    "dataset": "selection_indexes",
                    "description": "Dataset indicating for each axis the index of the selection applied to the given axis. -1 indicates that no selection is applied along that axis. The axis index ranges from -1 to n where -1 indicated global selection and n is the number of axes."
,
                    "dimensions": [
                        {
                            "axis": 0,
                            "dataset": null,
                            "description": "Integer index of the annotation",
                            "name": "annotation_index",
                            "optional": false,
                            "relationships": [],
                            "unit": null
                        },
                        {
                            "axis": 1,
                            "dataset": "axis_index",
                            "description": "Integer index of the axis",
                            "name": "axis_index",
                            "optional": false,
                            "relationships": [
                                {
                                    "attribute": "select_axis",
                                    "axis": null,
                                    "description": "Relationship documentation that each column of the selection_indexes dataset refers to a different selections_axis_ dataset",
                                    "optional": false,
                                    "prefix": null,
                                    "properties": null,
                                    "relationship_type": "order",
                                    "target": {
                                        "axis": null,
                                        "dataset": null,
                                        "filename": null,
                                        "global_path": null,
                                        "group": null,
                                        "prefix": "selections_axis_",
                                        "prefix_all": null
                                    }
                                }
                            ],
                            "unit": "index"
                        }
                    ],
                    "dimensions_fixed": true,
                    "optional": false,
                    "prefix": null,
                    "relationships": []
                },
                "selections": {
                    "attributes": [
                        {
                            "attribute": "axis",
                            "optional": false,
                            "prefix": null,
                            "value": null
                        }
                    ],
                    "dataset": null,
                    "description": "Datasets with all selections for the indicated axis
. Axis -1 indicates a global selection across all axes. One dataset per axis and one for global selection (-1) is mandatory.",
                    "dimensions": [
                        {
                            "axis": 0,
                            "dataset": null,
                            "description": "Integer index of the selection",
                            "name": "selection_index",
                            "optional": false,
                            "relationships": [],
```

```
                            "unit": null
                        }
                    ],
                    "dimensions_fixed": false,
                    "optional": false,
                    "prefix": "selections_axis_",
                    "relationships": []
                }
            },
            "description": "Managed group for storage of a collection of annotations. Multiple annotation collections may typically be associated with the same data object.",
            "group": null,
            "groups": {},
            "managed_objects": [],
            "optional": true,
            "prefix": "annotations_",
            "relationships": []
        }
    },
    "managed_objects": [],
    "optional": true,
    "prefix": "ephys_data_",
    "relationships": []
},
"ephys_data_processed_": {
    "attributes": [
        {
            "attribute": "format_type",
            "optional": false,
            "prefix": null,
            "value": "BrainDataEphysProcessed"
        },
        {
            "attribute": "format_description",
            "optional": false,
            "prefix": null,
            "value": "Managed group for storage of processed Ephys recordings."
        },
        {
            "attribute": "object_id",
            "optional": true,
            "prefix": null,
            "value": null
        },
        {
            "attribute": "format_specification",
            "optional": false,
            "prefix": null,
            "value": null
        }
    ],
    "datasets": {
        "ephys_data": {
            "attributes": [
                {
                    "attribute": "unit",
                    "optional": false,
                    "prefix": null,
                    "value": null
                },
                {
                    "attribute": "original_name",
                    "optional": true,
                    "prefix": null,
                    "value": null
                }
            ],
            "dataset": "processed_data",
            "description": "Dataset with the Ephys recordings data",
            "dimensions": [
                {
```

```
                "axis": 0,
                "dataset": "spatial_id",
                "description": "Id of the recording electrode",
                "name": "space",
                "optional": false,
                "relationships": [],
                "unit": "id"
            },
            {
                "axis": 1,
                "dataset": "time_axis",
                "description": "Sample time in ms",
                "name": "time",
                "optional": false,
                "relationships": [],
                "unit": "ms"
            },
            {
                "axis": 0,
                "dataset": "anatomy_name",
                "description": "Name of region location of the electrodes",
                "name": "space",
                "optional": true,
                "relationships": [],
                "unit": "region name"
            },
            {
                "axis": 0,
                "dataset": "anatomy_id",
                "description": "Integer id of the region location of the electrodes
",
                "name": "space",
                "optional": true,
                "relationships": [],
                "unit": "region id"
            },
            {
                "axis": 2,
                "dataset": "frequency_bands",
                "description": "Frequency bands of the channels",
                "name": "channels",
                "optional": true,
                "relationships": [],
                "unit": "Hz"
            },
            {
                "axis": 2,
                "dataset": "token_id",
                "description": "Integer Id of the token type",
                "name": "channels",
                "optional": true,
                "relationships": [],
                "unit": "token id"
            },
            {
                "axis": 2,
                "dataset": "token_name",
                "description": "Name of the token type",
                "name": "channels",
                "optional": true,
                "relationships": [],
                "unit": "token name"
            }
        ],
        "dimensions_fixed": true,
        "optional": false,
        "prefix": null,
        "primary": true,
        "relationships": []
    },
    "layout": {
        "attributes": [],
        "dataset": "layout",
```

```
                "description": "The physical layout of the electrodes.",
                "dimensions": [],
                "optional": true,
                "prefix": null,
                "relationships": []
            },
            "sampling_rate": {
                "attributes": [
                    {
                        "attribute": "unit",
                        "optional": false,
                        "prefix": null,
                        "value": "Hz"
                    }
                ],
                "dataset": "sampling_rate",
                "description": "Sampling rate in Hz",
                "dimensions": [],
                "optional": false,
                "prefix": null,
                "relationships": []
            }
        },
        "description": "Managed group for storage of processed Ephys recordings.",
        "group": null,
        "groups": {
            "annotations_": {
                "attributes": [
                    {
                        "attribute": "collection_description",
                        "optional": false,
                        "prefix": null,
                        "value": null
                    },
                    {
                        "attribute": "format_type",
                        "optional": false,
                        "prefix": null,
                        "value": "AnnotationDataGroup"
                    },
                    {
                        "attribute": "format_description",
                        "optional": false,
                        "prefix": null,
                        "value": "Managed group for storage of a collection of annotations. Multiple annotation collections may typically be associated with the same data object."
                    },
                    {
                        "attribute": "object_id",
                        "optional": true,
                        "prefix": null,
                        "value": null
                    },
                    {
                        "attribute": "format_specification",
                        "optional": false,
                        "prefix": null,
                        "value": null
                    }
                ],
                "datasets": {
                    "annotation_type_indexes": {
                        "attributes": [],
                        "dataset": "annotation_type_indexes",
                        "description": "Dataset indicating for each selection the index of the annotation type used. The annotation types are given in the annotation types dataset.",
                        "dimensions": [
                            {
                                "axis": 0,
                                "dataset": null,
                                "description": "Integer index into the annotation_types
```

```
array indicating the type of the annotation",
                    "name": "type_index",
                    "optional": false,
                    "relationships": [],
                    "unit": null
                }
            ],
            "dimensions_fixed": true,
            "optional": false,
            "prefix": null,
            "relationships": [
                {
                    "attribute": "indexes_annotation_types",
                    "axis": 0,
                    "description": "Relationship documentation that we are
storing references to annotation_types",
                    "optional": false,
                    "prefix": null,
                    "properties": null,
                    "relationship_type": "indexes",
                    "target": {
                        "axis": 0,
                        "dataset": "annotation_types",
                        "filename": null,
                        "global_path": null,
                        "group": null,
                        "prefix": null,
                        "prefix_all": null
                    }
                }
            ]
        },
        "annotation_types": {
            "attributes": [],
            "dataset": "annotation_types",
            "description": "List of all available annotation types",
            "dimensions": [
                {
                    "axis": 0,
                    "dataset": null,
                    "description": "Integer index of the type",
                    "name": "type_index",
                    "optional": false,
                    "relationships": [],
                    "unit": null
                }
            ],
            "dimensions_fixed": true,
            "optional": false,
            "prefix": null,
            "relationships": []
        },
        "data_object": {
            "attributes": [],
            "dataset": "data_object",
            "description": null,
            "dimensions": [],
            "optional": false,
            "prefix": null,
            "relationships": []
        },
        "descriptions": {
            "attributes": [],
            "dataset": "descriptions",
            "description": "Dataset with the annotation descriptions.",
            "dimensions": [
                {
                    "axis": 0,
                    "dataset": null,
                    "description": "Integer index of the annotation",
                    "name": "annotation_index",
                    "optional": false,
                    "relationships": [],
```

```
                    "unit": null
                }
            ],
            "dimensions_fixed": true,
            "optional": false,
            "prefix": null,
            "relationships": []
        },
        "properties": {
            "attributes": [
                {
                    "attribute": "name",
                    "optional": false,
                    "prefix": null,
                    "value": null
                }
            ],
            "dataset": null,
            "description": "Datasets with a particular property for all annotations.",
            "dimensions": [
                {
                    "axis": 0,
                    "dataset": null,
                    "description": "Integer index of the selection",
                    "name": "annotation_index",
                    "optional": false,
                    "relationships": [],
                    "unit": null
                }
            ],
            "dimensions_fixed": false,
            "optional": true,
            "prefix": "property_",
            "relationships": []
        },
        "selection_indexes": {
            "attributes": [],
            "dataset": "selection_indexes",
            "description": "Dataset indicating for each axis the index of the selection applied to the given axis. -1 indicates that no selection is applied along that axis. The axis index ranges from -1 to n where -1 indicated global selection and n is the number of axes.",
            "dimensions": [
                {
                    "axis": 0,
                    "dataset": null,
                    "description": "Integer index of the annotation",
                    "name": "annotation_index",
                    "optional": false,
                    "relationships": [],
                    "unit": null
                },
                {
                    "axis": 1,
                    "dataset": "axis_index",
                    "description": "Integer index of the axis",
                    "name": "axis_index",
                    "optional": false,
                    "relationships": [
                        {
                            "attribute": "select_axis",
                            "axis": null,
                            "description": "Relationship documentation that each column of the selection_indexes dataset refers to a different selections_axis_ dataset",
                            "optional": false,
                            "prefix": null,
                            "properties": null,
                            "relationship_type": "order",
                            "target": {
                                "axis": null,
                                "dataset": null,
                                "filename": null,
```

```
                                "global_path": null,
                                "group": null,
                                "prefix": "selections_axis_",
                                "prefix_all": null
                              }
                            }
                          ],
                          "unit": "index"
                        }
                      ],
                      "dimensions_fixed": true,
                      "optional": false,
                      "prefix": null,
                      "relationships": []
                    },
                    "selections": {
                      "attributes": [
                        {
                          "attribute": "axis",
                          "optional": false,
                          "prefix": null,
                          "value": null
                        }
                      ],
                      "dataset": null,
                      "description": "Datasets with all selections for the indicated axis. Axis -1 indicates a global selection across all axes. One dataset per axis and one for global selection (-1) is mandatory.",
                      "dimensions": [
                        {
                          "axis": 0,
                          "dataset": null,
                          "description": "Integer index of the selection",
                          "name": "selection_index",
                          "optional": false,
                          "relationships": [],
                          "unit": null
                        }
                      ],
                      "dimensions_fixed": false,
                      "optional": false,
                      "prefix": "selections_axis_",
                      "relationships": []
                    }
                  },
                  "description": "Managed group for storage of a collection of annotations. Multiple annotation collections may typically be associated with the same data object.",
                  "group": null,
                  "groups": {},
                  "managed_objects": [],
                  "optional": true,
                  "prefix": "annotations_",
                  "relationships": []
                }
              },
              "managed_objects": [],
              "optional": true,
              "prefix": "ephys_data_processed_",
              "relationships": []
            }
          },
          "managed_objects": [],
          "optional": false,
          "prefix": null,
          "relationships": []
        }
      },
      "managed_objects": [],
      "optional": false,
      "prefix": null,
      "relationships": []
    },
```

```
"descriptors": {
    "attributes": [
        {
            "attribute": "format_type",
            "optional": false,
            "prefix": null,
            "value": "BrainDataDescriptors"
        },
        {
            "attribute": "format_description",
            "optional": false,
            "prefix": null,
            "value": "Managed group for storage of a collection of brain data descriptors."
        },
        {
            "attribute": "object_id",
            "optional": true,
            "prefix": null,
            "value": null
        },
        {
            "attribute": "format_specification",
            "optional": false,
            "prefix": null,
            "value": null
        }
    ],
    "datasets": {},
    "description": "Managed group for storage of a collection of brain data descriptors.",
    "group": "descriptors",
    "groups": {
        "dynamic": {
            "attributes": [
                {
                    "attribute": "format_type",
                    "optional": false,
                    "prefix": null,
                    "value": "BrainDataDynamicDescriptors"
                },
                {
                    "attribute": "format_description",
                    "optional": false,
                    "prefix": null,
                    "value": "Managed group for storage of static descriptors."
                },
                {
                    "attribute": "object_id",
                    "optional": true,
                    "prefix": null,
                    "value": null
                },
                {
                    "attribute": "format_specification",
                    "optional": false,
                    "prefix": null,
                    "value": null
                }
            ],
            "datasets": {},
            "description": "Managed group for storage of static descriptors.",
            "group": "dynamic",
            "groups": {
                "_": {
                    "attributes": [
                        {
                            "attribute": "format_type",
                            "optional": false,
                            "prefix": null,
                            "value": "BrainDataMetadataGroup"
                        },
                        {
                            "attribute": "format_description",
                            "optional": false,
```

```
        "prefix": null,
        "value": "Metadata storage group"
    },
    {
        "attribute": "object_id",
        "optional": true,
        "prefix": null,
        "value": null
    },
    {
        "attribute": "format_specification",
        "optional": false,
        "prefix": null,
        "value": null
    }
],
"datasets": {
    "_": {
        "attributes": [
            {
                "attribute": "unit",
                "description": "Attribute describing the units of the metadata",
                "optional": true,
                "prefix": null,
                "value": null
            },
            {
                "attribute": "ontology",
                "description": "Attribute describing the ontology used for the metadata",
                "optional": true,
                "prefix": null,
                "value": null
            },
            {
                "attribute": "user_description",
                "description": "Attribute describing the purpose of the dataset",
                "optional": true,
                "prefix": null,
                "value": null
            },
            {
                "attribute": "format_type",
                "optional": false,
                "prefix": null,
                "value": "BrainDataMetadataDataset"
            },
            {
                "attribute": "format_description",
                "optional": false,
                "prefix": null,
                "value": "Metadata storage dataset"
            },
            {
                "attribute": "object_id",
                "optional": true,
                "prefix": null,
                "value": null
            },
            {
                "attribute": "format_specification",
                "optional": false,
                "prefix": null,
                "value": null
            }
        ],
        "dataset": null,
        "description": "Metadata storage dataset",
        "dimensions": [],
        "optional": true,
        "prefix": null,
        "relationships": []
```

```
                }
            },
            "description": "Metadata storage group",
            "group": null,
            "groups": {},
            "managed_objects": [],
            "optional": true,
            "prefix": null,
            "relationships": []
        }
    },
    "managed_objects": [],
    "optional": false,
    "prefix": null,
    "relationships": []
},
"static": {
    "attributes": [
        {
            "attribute": "format_type",
            "optional": false,
            "prefix": null,
            "value": "BrainDataStaticDescriptors"
        },
        {
            "attribute": "format_description",
            "optional": false,
            "prefix": null,
            "value": "Managed group for storage of static descriptors."
        },
        {
            "attribute": "object_id",
            "optional": true,
            "prefix": null,
            "value": null
        },
        {
            "attribute": "format_specification",
            "optional": false,
            "prefix": null,
            "value": null
        }
    ],
    "datasets": {},
    "description": "Managed group for storage of static descriptors.",
    "group": "static",
    "groups": {
        "_": {
            "attributes": [
                {
                    "attribute": "format_type",
                    "optional": false,
                    "prefix": null,
                    "value": "BrainDataMetadataGroup"
                },
                {
                    "attribute": "format_description",
                    "optional": false,
                    "prefix": null,
                    "value": "Metadata storage group"
                },
                {
                    "attribute": "object_id",
                    "optional": true,
                    "prefix": null,
                    "value": null
                },
                {
                    "attribute": "format_specification",
                    "optional": false,
                    "prefix": null,
                    "value": null
                }
```

```
    ],
    "datasets": {
        "_": {
            "attributes": [
                {
                    "attribute": "unit",
                    "description": "Attribute describing the units of the metadata",
                    "optional": true,
                    "prefix": null,
                    "value": null
                },
                {
                    "attribute": "ontology",
                    "description": "Attribute describing the ontology used for the metadata",
                    "optional": true,
                    "prefix": null,
                    "value": null
                },
                {
                    "attribute": "user_description",
                    "description": "Attribute describing the purpose of the dataset",
                    "optional": true,
                    "prefix": null,
                    "value": null
                },
                {
                    "attribute": "format_type",
                    "optional": false,
                    "prefix": null,
                    "value": "BrainDataMetadataDataset"
                },
                {
                    "attribute": "format_description",
                    "optional": false,
                    "prefix": null,
                    "value": "Metadata storage dataset"
                },
                {
                    "attribute": "object_id",
                    "optional": true,
                    "prefix": null,
                    "value": null
                },
                {
                    "attribute": "format_specification",
                    "optional": false,
                    "prefix": null,
                    "value": null
                }
            ],
            "dataset": null,
            "description": "Metadata storage dataset",
            "dimensions": [],
            "optional": true,
            "prefix": null,
            "relationships": []
        }
    },
    "description": "Metadata storage group",
    "group": null,
    "groups": {},
    "managed_objects": [],
    "optional": true,
    "prefix": null,
    "relationships": []
},
"instrument_": {
    "attributes": [
        {
            "attribute": "format_type",
            "optional": false,
```

```
                "prefix": null,
                "value": "BrainDataStaticDescriptorInstrument"
            },
            {
                "attribute": "format_description",
                "optional": false,
                "prefix": null,
                "value": "Group storing metadata about an instrument"
            },
            {
                "attribute": "object_id",
                "optional": true,
                "prefix": null,
                "value": null
            },
            {
                "attribute": "format_specification",
                "optional": false,
                "prefix": null,
                "value": null
            }
        ],
        "datasets": {
            "_": {
                "attributes": [
                    {
                        "attribute": "unit",
                        "description": "Attribute describing the units of the metadata",
                        "optional": true,
                        "prefix": null,
                        "value": null
                    },
                    {
                        "attribute": "ontology",
                        "description": "Attribute describing the ontology used for the metadata",
                        "optional": true,
                        "prefix": null,
                        "value": null
                    },
                    {
                        "attribute": "user_description",
                        "description": "Attribute describing the purpose of the dataset",
                        "optional": true,
                        "prefix": null,
                        "value": null
                    },
                    {
                        "attribute": "format_type",
                        "optional": false,
                        "prefix": null,
                        "value": "BrainDataMetadataDataset"
                    },
                    {
                        "attribute": "format_description",
                        "optional": false,
                        "prefix": null,
                        "value": "Metadata storage dataset"
                    },
                    {
                        "attribute": "object_id",
                        "optional": true,
                        "prefix": null,
                        "value": null
                    },
                    {
                        "attribute": "format_specification",
                        "optional": false,
                        "prefix": null,
                        "value": null
                    }
                ],
```

```
                                    "dataset": null,
                                    "description": "Metadata storage dataset",
                                    "dimensions": [],
                                    "optional": true,
                                    "prefix": null,
                                    "relationships": []
                                },
                                "layout_index": {
                                    "attributes": [],
                                    "dataset": "layout_index",
                                    "description": "Dataset describing the instrument layout of different recording channels.",
                                    "dimensions": [],
                                    "optional": true,
                                    "prefix": null,
                                    "relationships": []
                                },
                                "layout_locations": {
                                    "attributes": [],
                                    "dataset": "layout_locations",
                                    "description": "Dataset describing the location of the different recording channels in space.",
                                    "dimensions": [],
                                    "optional": true,
                                    "prefix": null,
                                    "relationships": []
                                },
                                "type": {
                                    "attributes": [],
                                    "dataset": "type",
                                    "description": "The type of instrument",
                                    "dimensions": [],
                                    "optional": false,
                                    "prefix": null,
                                    "relationships": []
                                }
                            },
                            "description": "Group storing metadata about an instrument",
                            "group": null,
                            "groups": {},
                            "managed_objects": [],
                            "optional": true,
                            "prefix": "instrument_",
                            "relationships": []
                        }
                    },
                    "managed_objects": [],
                    "optional": false,
                    "prefix": null,
                    "relationships": []
                }
            },
            "managed_objects": [],
            "optional": false,
            "prefix": null,
            "relationships": []
        }
    },
    "managed_objects": [],
    "optional": false,
    "prefix": "entry_",
    "relationships": []
}
```

3 Specification Document for brain.dataformat.brainformat

The specification document for a file format module—in this case the LBNL brain format—can be easily compiled directly from the given API modules. This allows developers to easily add new format classes and modules without having to maintain multiple documents.

```
import time
from brain.dataformat.spec import FormatDocument
import brain.dataformat.brainformat as brainformat
json_spec = json_spec = FormatDocument.from_api(module_object=brainformat).to_json(pretty=True)

print '**' + str(time.ctime(time.time())) + '**'
print json_spec
```

```
**Mon Sep 19 18:07:15 2016**
{
    "AnnotationDataGroup": {
        "attributes": [
            {
                "attribute": "collection_description",
                "optional": false,
                "prefix": null,
                "value": null
            }
        ],
        "datasets": {
            "annotation_type_indexes": {
                "attributes": [],
                "dataset": "annotation_type_indexes",
                "description": "Dataset indicating for each selection the index of the annotation type used. The annotation types are given in the annotation types dataset.",
                "dimensions": [
                    {
                        "axis": 0,
                        "dataset": null,
                        "description": "Integer index into the annotation_types array indicating the type of the annotation",
                        "name": "type_index",
                        "optional": false,
                        "relationships": [],
                        "unit": null
                    }
                ],
                "dimensions_fixed": true,
                "optional": false,
                "prefix": null,
                "relationships": [
                    {
                        "attribute": "indexes_annotation_types",
                        "axis": 0,
                        "description": "Relationship documentation that we are storing references to annotation_types",
                        "optional": false,
                        "prefix": null,
                        "properties": null,
                        "relationship_type": "indexes",
                        "target": {
                            "axis": 0,
                            "dataset": "annotation_types",
                            "filename": null,
                            "global_path": null,
                            "group": null,
                            "prefix": null,
                            "prefix_all": null
                        }
                    }
                ]
            },
            "annotation_types": {
                "attributes": [],
                "dataset": "annotation_types",
                "description": "List of all available annotation types",
                "dimensions": [
                    {
                        "axis": 0,
                        "dataset": null,
                        "description": "Integer index of the type",
                        "name": "type_index",
                        "optional": false,
                        "relationships": [],
                        "unit": null
                    }
                ],
                "dimensions_fixed": true,
                "optional": false,
                "prefix": null,
                "relationships": []
```

```
          },
          "data_object": {
            "attributes": [],
            "dataset": "data_object",
            "description": null,
            "dimensions": [],
            "optional": false,
            "prefix": null,
            "relationships": []
          },
          "descriptions": {
            "attributes": [],
            "dataset": "descriptions",
            "description": "Dataset with the annotation descriptions.",
            "dimensions": [
              {
                "axis": 0,
                "dataset": null,
                "description": "Integer index of the annotation",
                "name": "annotation_index",
                "optional": false,
                "relationships": [],
                "unit": null
              }
            ],
            "dimensions_fixed": true,
            "optional": false,
            "prefix": null,
            "relationships": []
          },
          "properties": {
            "attributes": [
              {
                "attribute": "name",
                "optional": false,
                "prefix": null,
                "value": null
              }
            ],
            "dataset": null,
            "description": "Datasets with a particular property for all annotations.",
            "dimensions": [
              {
                "axis": 0,
                "dataset": null,
                "description": "Integer index of the selection",
                "name": "annotation_index",
                "optional": false,
                "relationships": [],
                "unit": null
              }
            ],
            "dimensions_fixed": false,
            "optional": true,
            "prefix": "property_",
            "relationships": []
          },
          "selection_indexes": {
            "attributes": [],
            "dataset": "selection_indexes",
            "description": "Dataset indicating for each axis the index of the selection applied to the given axis. -1 indicates that no selection is applied along that axis. The axis index ranges from -1 to n where -1 indicated global selection and n is the number of axes.",
            "dimensions": [
              {
                "axis": 0,
                "dataset": null,
                "description": "Integer index of the annotation",
                "name": "annotation_index",
                "optional": false,
                "relationships": [],
                "unit": null
              },
```

```
                    {
                        "axis": 1,
                        "dataset": "axis_index",
                        "description": "Integer index of the axis",
                        "name": "axis_index",
                        "optional": false,
                        "relationships": [
                            {
                                "attribute": "select_axis",
                                "axis": null,
                                "description": "Relationship documentation that each column of the selection_indexes dataset refers to a different selections_axis_ dataset",
                                "optional": false,
                                "prefix": null,
                                "properties": null,
                                "relationship_type": "order",
                                "target": {
                                    "axis": null,
                                    "dataset": null,
                                    "filename": null,
                                    "global_path": null,
                                    "group": null,
                                    "prefix": "selections_axis_",
                                    "prefix_all": null
                                }
                            }
                        ],
                        "unit": "index"
                    }
                ],
                "dimensions_fixed": true,
                "optional": false,
                "prefix": null,
                "relationships": []
            },
            "selections": {
                "attributes": [
                    {
                        "attribute": "axis",
                        "optional": false,
                        "prefix": null,
                        "value": null
                    }
                ],
                "dataset": null,
                "description": "Datasets with all selections for the indicated axis. Axis -1 indicates a global selection across all axes. One dataset per axis and one for global selection (-1) is mandatory.",
                "dimensions": [
                    {
                        "axis": 0,
                        "dataset": null,
                        "description": "Integer index of the selection",
                        "name": "selection_index",
                        "optional": false,
                        "relationships": [],
                        "unit": null
                    }
                ],
                "dimensions_fixed": false,
                "optional": false,
                "prefix": "selections_axis_",
                "relationships": []
            }
        },
        "description": "Managed group for storage of a collection of annotations. Multiple annotation collections may typically be associated with the same data object.",
        "group": null,
        "groups": {},
        "managed_objects": [],
        "optional": true,
        "prefix": "annotations_",
        "relationships": []
```

```
    },
    "BrainDataCollection": {
        "attributes": [],
        "datasets": {},
        "description": "Container for storing a collection of related datasets, e.g. from a single session or modality",
        "group": null,
        "groups": {},
        "managed_objects": [
            {
                "format_type": "BrainDataEphys",
                "optional": true
            },
            {
                "format_type": "BrainDataEphysProcessed",
                "optional": true
            }
        ],
        "optional": true,
        "prefix": "collection_",
        "relationships": []
    },
    "BrainDataData": {
        "attributes": [],
        "datasets": {},
        "description": "Managed group for storage of brain data (internal and external).",
        "group": "data",
        "groups": {},
        "managed_objects": [
            {
                "format_type": "BrainDataInternalData",
                "optional": false
            },
            {
                "format_type": "BrainDataExternalData",
                "optional": false
            }
        ],
        "optional": false,
        "prefix": null,
        "relationships": []
    },
    "BrainDataDescriptors": {
        "attributes": [],
        "datasets": {},
        "description": "Managed group for storage of a collection of brain data descriptors.",
        "group": "descriptors",
        "groups": {},
        "managed_objects": [
            {
                "format_type": "BrainDataStaticDescriptors",
                "optional": false
            },
            {
                "format_type": "BrainDataDynamicDescriptors",
                "optional": false
            }
        ],
        "optional": false,
        "prefix": null,
        "relationships": []
    },
    "BrainDataDynamicDescriptors": {
        "attributes": [],
        "datasets": {},
        "description": "Managed group for storage of static descriptors.",
        "group": "dynamic",
        "groups": {},
        "managed_objects": [
            {
                "format_type": "BrainDataMetadataGroup",
                "optional": true
            }
```

```
        ],
        "optional": false,
        "prefix": null,
        "relationships": []
    },
    "BrainDataEphys": {
        "attributes": [],
        "datasets": {
            "ephys_data": {
                "attributes": [
                    {
                        "attribute": "unit",
                        "optional": false,
                        "prefix": null,
                        "value": "Volt"
                    }
                ],
                "dataset": "raw_data",
                "description": "Dataset with the Ephys recordings data",
                "dimensions": [
                    {
                        "axis": 0,
                        "dataset": "electrode_id",
                        "description": "Id of the recording electrode",
                        "name": "space",
                        "optional": false,
                        "relationships": [],
                        "unit": "id"
                    },
                    {
                        "axis": 1,
                        "dataset": "time_axis",
                        "description": "Sample time in ms",
                        "name": "time",
                        "optional": false,
                        "relationships": [],
                        "unit": "ms"
                    },
                    {
                        "axis": 0,
                        "dataset": "anatomy_name",
                        "description": "Name of region location of the electrodes",
                        "name": "space",
                        "optional": true,
                        "relationships": [],
                        "unit": "region name"
                    },
                    {
                        "axis": 0,
                        "dataset": "anatomy_id",
                        "description": "Integer id of the region location of the electrodes",
                        "name": "space",
                        "optional": true,
                        "relationships": [],
                        "unit": "region id"
                    }
                ],
                "dimensions_fixed": true,
                "optional": false,
                "prefix": null,
                "primary": true,
                "relationships": []
            },
            "layout": {
                "attributes": [],
                "dataset": "layout",
                "description": "The physical layout of the electrodes.",
                "dimensions": [],
                "optional": true,
                "prefix": null,
                "relationships": []
            },
            "sampling_rate": {
```

```
                "attributes": [
                    {
                        "attribute": "unit",
                        "optional": false,
                        "prefix": null,
                        "value": "Hz"
                    }
                ],
                "dataset": "sampling_rate",
                "description": "Sampling rate in Hz",
                "dimensions": [],
                "optional": false,
                "prefix": null,
                "relationships": []
            }
        },
        "description": "Managed group for storage of raw Ephys recordings.",
        "group": null,
        "groups": {},
        "managed_objects": [
            {
                "format_type": "AnnotationDataGroup",
                "optional": true
            }
        ],
        "optional": false,
        "prefix": "ephys_data_",
        "relationships": []
    },
    "BrainDataEphysProcessed": {
        "attributes": [],
        "datasets": {
            "ephys_data": {
                "attributes": [
                    {
                        "attribute": "unit",
                        "optional": false,
                        "prefix": null,
                        "value": null
                    },
                    {
                        "attribute": "original_name",
                        "optional": true,
                        "prefix": null,
                        "value": null
                    }
                ],
                "dataset": "processed_data",
                "description": "Dataset with the Ephys recordings data",
                "dimensions": [
                    {
                        "axis": 0,
                        "dataset": "spatial_id",
                        "description": "Id of the recording electrode",
                        "name": "space",
                        "optional": false,
                        "relationships": [],
                        "unit": "id"
                    },
                    {
                        "axis": 1,
                        "dataset": "time_axis",
                        "description": "Sample time in ms",
                        "name": "time",
                        "optional": false,
                        "relationships": [],
                        "unit": "ms"
                    },
                    {
                        "axis": 0,
                        "dataset": "anatomy_name",
                        "description": "Name of region location of the electrodes",
                        "name": "space",
```

```
                    "optional": true,
                    "relationships": [],
                    "unit": "region name"
                },
                {
                    "axis": 0,
                    "dataset": "anatomy_id",
                    "description": "Integer id of the region location of the electrodes",
                    "name": "space",
                    "optional": true,
                    "relationships": [],
                    "unit": "region id"
                },
                {
                    "axis": 2,
                    "dataset": "frequency_bands",
                    "description": "Frequency bands of the channels",
                    "name": "channels",
                    "optional": true,
                    "relationships": [],
                    "unit": "Hz"
                },
                {
                    "axis": 2,
                    "dataset": "token_id",
                    "description": "Integer Id of the token type",
                    "name": "channels",
                    "optional": true,
                    "relationships": [],
                    "unit": "token id"
                },
                {
                    "axis": 2,
                    "dataset": "token_name",
                    "description": "Name of the token type",
                    "name": "channels",
                    "optional": true,
                    "relationships": [],
                    "unit": "token name"
                }
            ],
            "dimensions_fixed": true,
            "optional": false,
            "prefix": null,
            "primary": true,
            "relationships": []
        },
        "layout": {
            "attributes": [],
            "dataset": "layout",
            "description": "The physical layout of the electrodes.",
            "dimensions": [],
            "optional": true,
            "prefix": null,
            "relationships": []
        },
        "sampling_rate": {
            "attributes": [
                {
                    "attribute": "unit",
                    "optional": false,
                    "prefix": null,
                    "value": "Hz"
                }
            ],
            "dataset": "sampling_rate",
            "description": "Sampling rate in Hz",
            "dimensions": [],
            "optional": false,
            "prefix": null,
            "relationships": []
        }
    },
```

```
        "description": "Managed group for storage of processed Ephys recordings.",
        "group": null,
        "groups": {},
        "managed_objects": [
            {
                "format_type": "AnnotationDataGroup",
                "optional": true
            }
        ],
        "optional": false,
        "prefix": "ephys_data_processed_",
        "relationships": []
    },
    "BrainDataExternalData": {
        "attributes": [],
        "datasets": {},
        "description": "Managed group for storage of external data related to the internal brain data.",
        "group": "external",
        "groups": {},
        "managed_objects": [
            {
                "format_type": "BrainDataStimulus",
                "optional": true
            }
        ],
        "optional": false,
        "prefix": null,
        "relationships": []
    },
    "BrainDataFile": {
        "attributes": [],
        "datasets": {},
        "description": "Managed BRAIN file.",
        "file_extension": ".h5",
        "file_prefix": null,
        "group": null,
        "groups": {},
        "managed_objects": [
            {
                "format_type": "BrainDataData",
                "optional": false
            },
            {
                "format_type": "BrainDataDescriptors",
                "optional": false
            }
        ],
        "optional": false,
        "prefix": "entry_",
        "relationships": []
    },
    "BrainDataInternalData": {
        "attributes": [],
        "datasets": {},
        "description": "Managed group for storage of a collection of internal brain data.",
        "group": "internal",
        "groups": {},
        "managed_objects": [
            {
                "format_type": "BrainDataEphys",
                "optional": true
            },
            {
                "format_type": "BrainDataEphysProcessed",
                "optional": true
            },
            {
                "format_type": "BrainDataCollection",
                "optional": true
            }
        ],
        "optional": false,
```

```
        "prefix": null,
        "relationships": []
    },
    "BrainDataMetadataDataset": {
        "attributes": [
            {
                "attribute": "unit",
                "description": "Attribute describing the units of the metadata",
                "optional": true,
                "prefix": null,
                "value": null
            },
            {
                "attribute": "ontology",
                "description": "Attribute describing the ontology used for the metadata",
                "optional": true,
                "prefix": null,
                "value": null
            },
            {
                "attribute": "user_description",
                "description": "Attribute describing the purpose of the dataset",
                "optional": true,
                "prefix": null,
                "value": null
            }
        ],
        "dataset": null,
        "description": "Metadata storage dataset",
        "dimensions": [],
        "optional": false,
        "prefix": null,
        "relationships": []
    },
    "BrainDataMetadataGroup": {
        "attributes": [],
        "datasets": {},
        "description": "Metadata storage group",
        "group": null,
        "groups": {},
        "managed_objects": [
            {
                "format_type": "BrainDataMetadataDataset",
                "optional": true
            }
        ],
        "optional": false,
        "prefix": null,
        "relationships": []
    },
    "BrainDataMultiFile": {
        "attributes": [],
        "datasets": {},
        "description": "Container file used to organize multiple BrainDataFile objects into a larger data collection, e.g., to create a collection of recording session or experiments allowing users to more seamlessly interact with many related files.",
        "file_extension": ".h5",
        "file_prefix": null,
        "group": null,
        "groups": {},
        "managed_objects": [
            {
                "format_type": "BrainDataFile",
                "optional": true
            }
        ],
        "optional": false,
        "prefix": null,
        "relationships": []
    },
    "BrainDataStaticDescriptorInstrument": {
        "attributes": [],
        "datasets": {
```

```
            "layout_index": {
                "attributes": [],
                "dataset": "layout_index",
                "description": "Dataset describing the instrument layout of different recording channels.",
                "dimensions": [],
                "optional": true,
                "prefix": null,
                "relationships": []
            },
            "layout_locations": {
                "attributes": [],
                "dataset": "layout_locations",
                "description": "Dataset describing the location of the different recording channels in space.",
                "dimensions": [],
                "optional": true,
                "prefix": null,
                "relationships": []
            },
            "type": {
                "attributes": [],
                "dataset": "type",
                "description": "The type of instrument",
                "dimensions": [],
                "optional": false,
                "prefix": null,
                "relationships": []
            }
        },
        "description": "Group storing metadata about an instrument",
        "group": null,
        "groups": {},
        "managed_objects": [
            {
                "format_type": "BrainDataMetadataDataset",
                "optional": true
            }
        ],
        "optional": false,
        "prefix": "instrument_",
        "relationships": []
    },
    "BrainDataStaticDescriptors": {
        "attributes": [],
        "datasets": {},
        "description": "Managed group for storage of static descriptors.",
        "group": "static",
        "groups": {},
        "managed_objects": [
            {
                "format_type": "BrainDataStaticDescriptorInstrument",
                "optional": true
            },
            {
                "format_type": "BrainDataMetadataGroup",
                "optional": true
            }
        ],
        "optional": false,
        "prefix": null,
        "relationships": []
    },
    "BrainDataStimulus": {
        "attributes": [],
        "datasets": {
            "type": {
                "attributes": [],
                "dataset": "type",
                "description": "The type of stimulus",
                "dimensions": [],
                "optional": false,
                "prefix": null,
```

```
                "relationships": []
            }
        },
        "description": "Group stroing metadata about a stimulus",
        "group": null,
        "groups": {},
        "managed_objects": [
            {
                "format_type": "BrainDataMetadataDataset",
                "optional": true
            }
        ],
        "optional": false,
        "prefix": "stimulus_",
        "relationships": []
    },
    "ManagedObjectFile": {
        "attributes": [],
        "datasets": {},
        "description": "Container file used for external storage of managed objects. This container file is used to allow modular files where different components of a file are stored in separate files that are linked viahard links.",
        "file_extension": ".h5",
        "file_prefix": null,
        "group": "/",
        "groups": {},
        "managed_objects": [
            {
                "format_type": "ManagedObject",
                "optional": true
            }
        ],
        "optional": false,
        "prefix": null,
        "relationships": []
    }
}
```

4 License & Copyright

4.1 License

BrainFormat Copyright (c) 2014, The Regents of the University of California, through Lawrence Berkeley National Laboratory (subject to receipt of any required approvals from the U.S. Dept. of Energy). All rights reserved.

Redistribution and use in source and binary forms, with or without modification, are permitted provided that the following conditions are met:

1. Redistributions of source code must retain the above copyright notice, this list of conditions and the following disclaimer.
2. Redistributions in binary form must reproduce the above copyright notice, this list of conditions and the following disclaimer in the documentation and/or other materials provided with the distribution.
3. Neither the name of the University of California, Lawrence Berkeley National Laboratory, U.S. Dept. of Energy nor the names of its contributors may be used to endorse or promote products derived from this software without specific prior written permission.

THIS SOFTWARE IS PROVIDED BY THE COPYRIGHT HOLDERS AND CONTRIBUTORS “AS IS” AND ANY EXPRESS OR IMPLIED WARRANTIES, INCLUDING, BUT NOT LIMITED TO, THE IMPLIED WARRANTIES OF MERCHANTABILITY AND FITNESS FOR A PARTICULAR PURPOSE ARE DISCLAIMED. IN NO EVENT SHALL THE COPYRIGHT OWNER OR CONTRIBUTORS BE LIABLE FOR ANY DIRECT, INDIRECT, INCIDENTAL, SPECIAL, EXEMPLARY, OR CONSEQUENTIAL DAMAGES (INCLUDING, BUT NOT LIMITED TO, PROCUREMENT OF SUBSTITUTE GOODS OR SERVICES; LOSS OF USE, DATA, OR PROFITS; OR BUSINESS INTERRUPTION) HOWEVER CAUSED AND ON ANY THEORY OF LIABILITY, WHETHER IN CONTRACT, STRICT LIABILITY, OR TORT (INCLUDING NEGLIGENCE OR OTHERWISE) ARISING IN ANY WAY OUT OF THE USE OF THIS SOFTWARE, EVEN IF ADVISED OF THE POSSIBILITY OF SUCH DAMAGE.

You are under no obligation whatsoever to provide any bug fixes, patches, or upgrades to the features, functionality or performance of the source code (“Enhancements”) to anyone; however, if you choose to make your Enhancements available either publicly, or directly to Lawrence Berkeley National Laboratory, without imposing a separate written license agreement for such Enhancements, then you hereby grant the following license: a non-exclusive, royalty-free perpetual license to install, use, modify, prepare derivative works, incorporate into other computer software, distribute, and sublicense such enhancements or derivative works thereof, in binary and source code form.

4.2 Copyright

BrainFormat Copyright (c) 2014, The Regents of the University of California, through Lawrence Berkeley National Laboratory (subject to receipt of any required approvals from the U.S. Dept. of Energy). All rights reserved.

If you have questions about your rights to use or distribute this software, please contact Berkeley Lab’s Innovation & Partnerships Office at IPO@lbl.gov referring to ” BrainFormat (LBNL Ref 2015-020).”

NOTICE. This software was developed under funding from the U.S. Department of Energy. As such, the U.S. Government has been granted for itself and others acting on its behalf a paid-up, nonexclusive, irrevocable, worldwide license in the Software to reproduce, prepare derivative works, and perform publicly and display publicly. Beginning five (5) years after the date permission to assert copyright is obtained from the U.S. Department of Energy, and subject to any subsequent five (5) year renewals, the U.S. Government is granted for itself and others acting on its behalf a paid-up, nonexclusive, irrevocable, worldwide license in the Software to reproduce, prepare derivative works, distribute copies to the public, perform publicly and display publicly, and to permit others to do so.

SUPPLEMENT 2: BRAINDATAEPHYS AUTO-EXPAND-MODE EXAMPLE

The *auto-expand-data* feature of `BrainDataEphys` is designed to facilitate the incremental acquisition of voltage recordings over time. Using this feature we can, for example do the following:

```
>>> from brain.dataformat.brainformat import BrainDataFile, BrainDataEphys
>>> import numpy
>>> brainfile = BrainDataFile.create('testfile.h5')   # Create the file and initialize the data hierarchy
>>> internal_data = brainfile.data().internal()        # Get the managed object for storing internal data
>>> ecog_data = BrainDataEphys.create(parent_object=internal_data ,     # Add to /data/internal
                                     ephys_data_shape=(32,0),           # Empty recording for 32 electrodes
                                     ephys_data_type='f',               # Store float data values
                                     chunks=True)                      # Store the data using chunking
>>> ecog_data.set_auto_expand(True)                                    # Enable auto expansion
>>> ecog_data[:, 0:1000] = numpy.arange(32*1000).reshape(32,1000)      # Add new data
```

Note when adding the new data, the shape of our electrophysiology dataset is automatically expanded to 32×1000 and all one-dimensional dimension-scales that are associated with the time axis are automatically expanded to match the new data shape so that we can also conveniently update the data of dimension scales without having to resize the datasets manually. As the above example illustrates, the `BrainDataEphys` API provides a convenient interface that allows us to directly interact with the primary raw data via standard array-based data selection operations while auxiliary data, e.g., the sampling rate, layout, etc., can be easily retrieved via corresponding access functions or key-based data selection (similar to Python dictionaries).

SUPPLEMENT 3: APPLICATION TO ELECTROCORTICOGRAPHY (ECOG) DURING SPEECH PRODUCTION

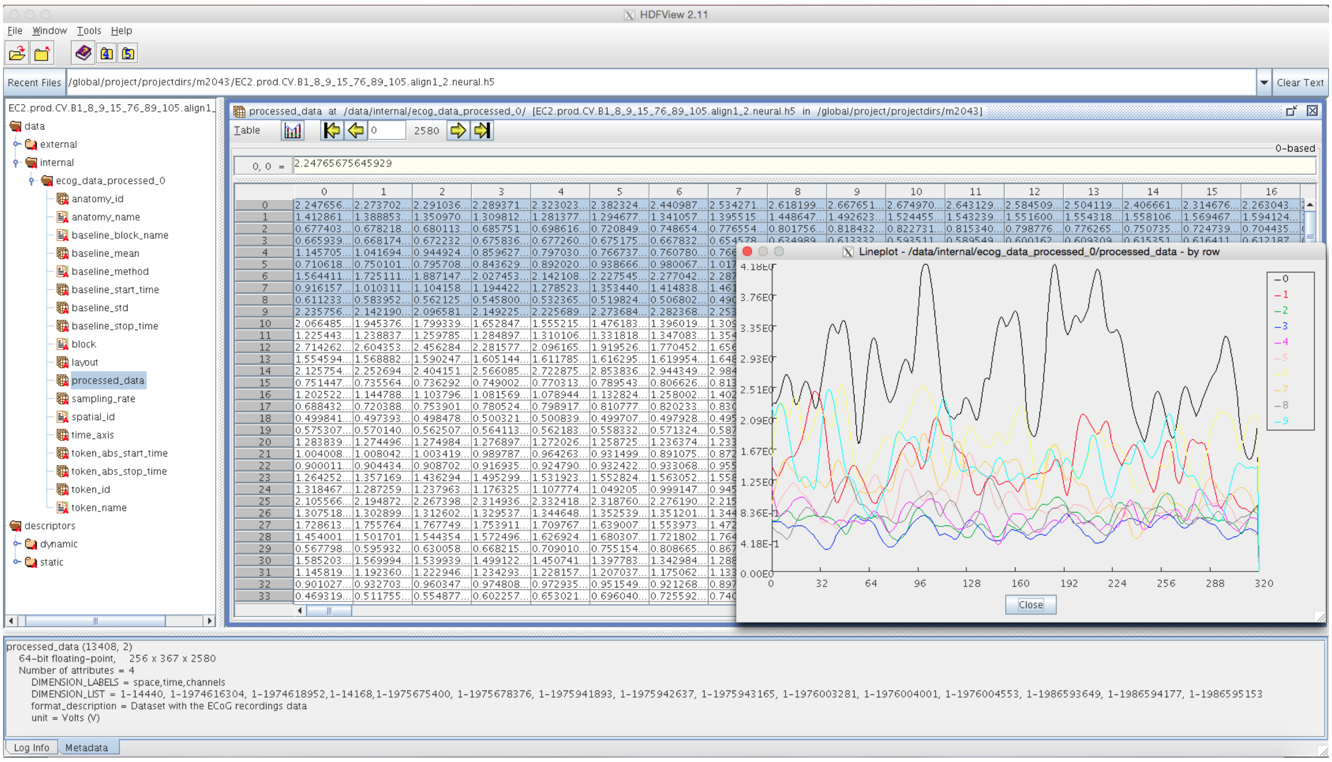

Figure 1. Example HDFView visualization of a BRAINformat file. **Left:** Tree view of the basic data hierarchy. **Right:** Table view of a processed ECoG dataset and curve plot of the first 10 waveforms. **Bottom:** Summary of properties and attributes of the processed ECoG data array.

Figure 1 shows an example BRAINformat file of electrocorticography (ECoG) data collected from neurosurgical patients during speech production. The file is visualized in HDFView[1], a standard HDF5 file browser. The example view shows a processed dataset in which the data has been processed to extract specific, fixed-length speech events/features from the data. As a result the data is reorganized as a three-dimensional array of $space \times time \times event$. In addition to the *spatial_id* electrode id's of the recording device and corresponding `layout` information, the space dimension is further characterized by `anatomy_id` and `anatomy_name` dimensions scales describing the anatomical name and integer id of the spatial region where each electrode is located. The event dimension is further described by dimension scales with the name and id of the tokens (`token_name` and `token_id`) as well as the absolute start and stop time of each token (`token_abs_start_time` and `token_abs_stop_time`). The tree view on the left shows the file structure, including all datasets associated with the `/data/internal/ecog_data_processed_#` group. The table view on the right then shows the contents of the primary `processed_data` dataset and the curve plot shows the voltage signal over time for a select set of tokens/electrodes. The properties view at the bottom then shows the shape, data type, and attributes associated with the main dataset.

[1] The HDF Group. HDFView, 2006 2015. [ONLINE] `http://www.hdfgroup.org/products/java/hdfview/`
